# Genome-wide CG hypomethylation of the *Arabidopsis* ecotype Cvi linked to structural variation and RNAi at the *VIM4*–*VIM2* locus

**DOI:** 10.1073/pnas.2603682123

**Published:** 2026-05-19

**Authors:** Sang-Yoon Shin, Minsu Park, Jaehoon Lee, Seunga Lee, Jennifer M. Frost, Robert L. Fischer, Yeonhee Choi, Chanseok Shin

**Affiliations:** ^a^Research Institute of Agriculture and Life Sciences, and Plant Genomics and Breeding Institute, Seoul National University, Seoul 08826, Korea; ^b^Research Center for Plant Plasticity, Seoul National University, Seoul 08826, Korea; ^c^Department of Agricultural Biotechnology, Seoul National University, Seoul 08826, Korea; ^d^Department of Biological Sciences, Seoul National University, Seoul 08826, Korea; ^e^Department of Medical and Molecular Genetics, King’s College London, Great Maze Pond, London SE1 9RT, United Kingdom; ^f^Department of Plant and Microbial Biology, University of California, Berkeley, CA 94720

**Keywords:** whole genome methylation, *Arabidopsis* ecotype, small RNA, natural variation

## Abstract

The Cape Verde Island (Cvi)-0 ecotype, a relict lineage of *Arabidopsis thaliana*, is characterized by exceptionally low CG methylation levels genome-wide. Numerous population-level and molecular studies have highlighted links between this epigenomic feature, phenotypic traits and environmental factors; however, the molecular mechanism underlying Cvi-0’s distinctive methylation profile remains unknown. Using multiomics comparisons between Cvi-0 and Col-0, we identified Cvi-specific small-RNAs derived from an inverted repeat (IR) formed by two inverted-orientation DNA methylation pathway genes, *VARIANT IN METHYLATION 2* and *4* (*VIM2* and *VIM4*). An intergenic 2.8-kb deletion brings these genes closer in Cvi-0, enabling read-through transcription and formation of chimeric transcripts. Targeted 5′ RACE and WGBS showed that the resulting siRNAs mediate both posttranscriptional gene silencing and local DNA hypermethylation *in cis* and *trans,* reducing the expression of the *VIM1* gene, which is required for methyltransferase activity in *A.thaliana*. Comparative analysis across *A. thaliana* ecotypes and related species revealed substantial structural polymorphism at the *VIM4*–*VIM2* IR locus, likely shaped by nonallelic homologous recombination—facilitated by local palindromic structures, intronic repeats, and transposable element activity. Notably, the Cvi-0 *VIM4*–*VIM2* IR configuration was unique, suggesting it is a rare, lineage-specific natural variant. Taken together, natural variation at the *VIM4*–*VIM2* palindrome locus, uniquely found in Cvi-0, appears to be the causal variant driving siRNA biogenesis and down-regulation of the methyltransferase pathway. This distinctive regulatory mechanism is likely to have contributed to long-term reduction of CG methylation in Cvi-0, potentially maintained over millennia due to its geographical isolation from the mainland.

## Significance

DNA methylation is required for genome stability and regulation of genes and transposons in many eukaryotes. In *Arabidopsis thaliana*, DNA methylation is required for developmental programming, yet profiles vary dramatically according to geography and climate. The commonly used Cvi-0 ecotype, discovered and collected from the Cape Verde Islands archipelago, exhibits deep genomic DNA hypomethylation, the origins of which are unknown. Here, we show that Cvi-specific structural variation at methylation pathway genes (*VIM4* and *VIM2*), results in small interfering RNA production and silencing of *VIM1*. We outline substantial structural polymorphism within the *VIM4*–*VIM2* region across many *A. thaliana* ecotypes, but find that the locus structure at Cvi-0 is unique. These observations implicate structural genetic changes in epigenetic variation and adaptation.

The *Arabidopsis thaliana* (*A*. *thaliana*) Cape Verde Island (Cvi)-0 ecotype was originally discovered and collected from the Cape Verde Islands archipelago around forty years ago, and is now one of the most widely studied accessions (1). Cvi-0 is a so-called relict lineage of *A.thaliana*, likely surviving the Pleistocene glacial period in the North-West African refugia, colonizing Cape Verde 5 to 7,000 years ago (2). DNA methylation variation across *A.thaliana* accessions is strongly associated with geographical location and climate, indicating that this epigenetic mark plays a role in evolutionary adaptation (3). Following a genetic bottleneck due to geological isolation in an extremely arid climate, Cvi exhibits distinctive genetic and phenotypic traits, including exceptionally low genome-wide DNA methylation at CG sites (3–5). In fact, Cvi has the lowest level of gene-body methylation (gbM) among all *A.thaliana* accessions, and the second-lowest number of methylated genes (3). As such, this natural accession has become a valuable model for studying phenotypic, environmental, and genetic crosstalk, as well as developmental and adaptive evolution in *A. thaliana* (2, 4, 6, 7). However, the origin of genome-wide hypomethylation in Cvi-0 remains unexplained.

METHYLTRANSFERASE 1 (MET1), a DNMT1 homologue, is a key factor in establishing, maintaining and inheriting mCG in *Arabidopsis*, but requires VARIANT IN METHYLATION (VIM) proteins for maintenance methylation activity at CG sites (8–10). VIM proteins share structural homology with mammalian Ubiquitin-like with PHD and RING Finger 1 (UHRF1, also known as Np95/ICBP90), featuring conserved SRA (Set and RING Associated) domains for preferential binding to hemimethylated CG and a RING domain for E3 ubiquitin ligase activity (10). In *Arabidopsis*, six VIM family proteins are encoded across the genome. VIMs1-5, contain a PHD domain, two RING domains, and an SRA domain, enabling them to bind methylated DNA (5mC) and recruit chromatin modifiers (8, 10, 11). In contrast, VIM6 (also known as ORTHRUS-LIKE 1) retains only the SRA domain and a single RING domain, which exhibits in vitro E3 ubiquitin ligase activity similar to that of VIM1-VIM3 (10). In Col-0, VIM1, 2, and 3 are essential for maintaining CG methylation at centromeric repeats and transposons enriched in heterochromatin regions, as well as at genic regions, in collaboration with MET1 (9, 12, 13). *vim1/2/3* mutants exhibit genome-wide CG/CHG hypomethylation, associated with derepression of transposable elements and protein-coding genes (14). *VIM* genes are also known to be functionally redundant—single mutants show minimal phenotypic effects, while *vim1/2/3* displays severe developmental defects, including delayed flowering due to reactivation of *FWA* (8). *vim1/2/3* mutants have also been reported to have reduced levels of repressive histone marks H3K9me2 and H3K27me3, and increased levels of active marks H3K4me3 and H3K9ac (12). Moreover, the E3 ubiquitin ligase activity of VIM1, mediated by its RING domains, links ubiquitination pathways to epigenetic silencing (10, 12).

Here, we employed comparative multiomic analyses of *Arabidopsis* ecotypes Col-0 and Cvi-0, and identified distinct small RNA populations detected exclusively in Cvi-0 allele-containing plants. We found the origin of the sRNAs to be loci encoding *VIM* family genes, *VIM2_Cvi_* and *VIM4_Cvi_*, and *PAI* family genes, *PAI1_Cvi_* and *PAI4_Cvi_*. Pairwise comparison of these regions between genomes of Col-0 and Cvi-0 revealed two inverted repeats (IR) unique to the Cvi-0 genome: a head-to-head orientation between *VIM4_Cvi_*–*VIM2_Cvi_*, identified in this study, and a tail-to-tail orientation between *PAI4_Cvi_*–*PAI1_Cvi_*, previously reported (15, 16). These palindromic configurations are strongly implicated in siRNA production, likely driven by the transcription of alternative isoforms that form near-perfect stem-loop RNA structures specifically in Cvi-0. siRNA expression at *VIM4_Cvi_*–*VIM2_Cvi_* caused RdDM-mediated non-CG methylation at this locus, and posttranscriptional down-regulation of *VIM1* expression. We also found that the *VIM4_Cvi_*–*VIM2_Cvi_* locus exhibits extensive polymorphism among Arabidopsis accessions, attributable to its complex and repetitive sequence. Our findings uncover a previously unrecognized, Cvi-0-specific siRNA-driven regulatory layer that likely contributes to the uniquely low genome-wide CG methylation levels in these plants.

## Results

### Cvi-Specific sRNA Production from *VIM* and *PAI* Family Orthologs.

**Fig. 1.**
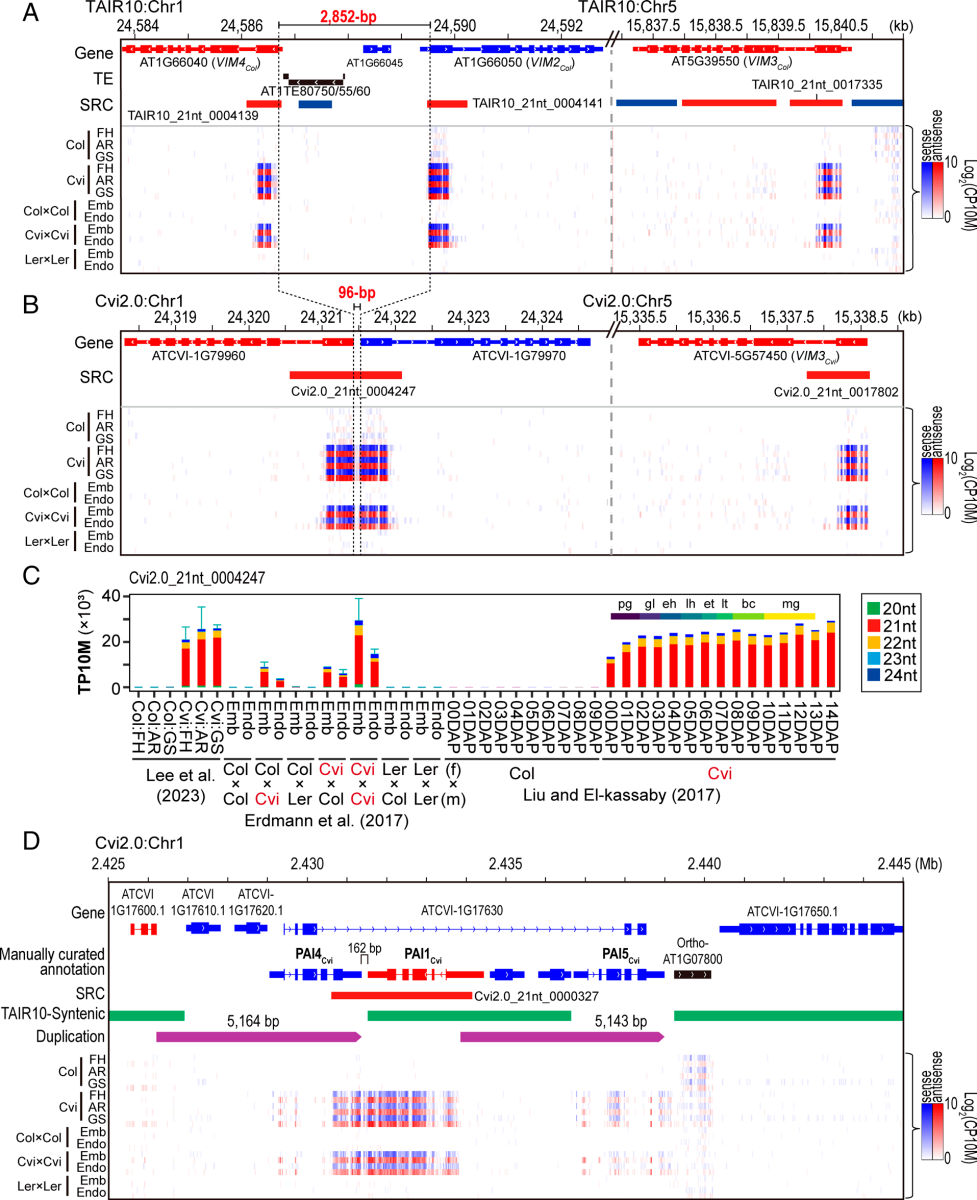
Cvi-allele-dependent presence of sRNA populations at *VIM* and *PAI* family genes. (*A* and *B*) Distribution of sRNAs across three *VIM* family genes-loci in TAIR10 (*A*) and in Cvi2.0 (*B*). Dotted lines indicate *VIM2* or *VIM4*’s start codon, and red-colored numbers between two dotted lines indicate the genomic distance. Log_2_-transformed CP10M-normalized sRNA abundance is represented as a heatmap. For the sRNA abundance heatmap, sRNAs mapped in the sense orientation are blue, and sRNAs mapped in antisense are red. FH; freshly harvested, AR; after-ripening GS; germination-stimulated, Emb; embryo, Endo; endosperm. (*C*) Spatiotemporal expression patterns and size composition of sRNAs produced from the region spanning the 1st exon of two *VIM* family homologs identified from Cvi’s reference genome, Cvi2.0. The height of the bar graph represents the mean TP10M-normalized sRNA abundance measured across sRNA-Seq replicates, and the error bars represent the SD of TP10M values for the total abundance of 20 to 24 nt sRNAs across replicates. Each color in the bar graph represents the abundance of each sRNA size. For the datasets from Erdmann et al., the maternal ecotype comes above the “×” sign [“(f)” for female] and the paternal ecotype comes below the “×” sign [“(m)” for male]. Datasets produced by Liu and El-Kassaby (SRP072220) did not include replicates; therefore, no error bars are drawn. DAP; days after pollination, Ler; Lansberg erecta, pg; preglobular, gl; globular, eh; early heart, lh; late heart, lt; late torpedo, bc; bent cotyledon, mg; mature green. (*D*) sRNA distribution across Cvi’s genomic region encoding three *PAI* family genes. “Manually curated annotation” track represents annotation of genes that are manually defined on the basis of sequence similarity in this study. “TAIR10-Syntenic” track represents regions that are syntenic between Cvi’s genome and the TAIR10 reference genome. “Duplication” track represents two ~5.1-kb loci exhibiting high sequence homology between them.

Using sRNA-seq data from our previous genome-wide analysis of seeds from two *Arabidopsis* ecotypes, Col and Cvi (5), we identified numerous genomic loci that predominantly produce 21-nt sRNA species and exhibit differential expression between the two ecotypes. Among these, three sRNA clusters located within each first exon of the three *VIM* family genes—*VIM4_Col_* (AT1G66040), *VIM2_Col_* (AT1G66050), and *VIM3_Col_* (AT5G39550)—showed Cvi-specific high sRNA abundance across all developing seed samples (Fig. 1*A* and Dataset S1). This Cvi-specific sRNA expression was validated using publicly available sRNA-Seq datasets from Col, Cvi, and Ler (Fig. 1*A* and *SI Appendix,* Fig. S1 *A*–*C*) (17, 18). Notably, sRNA profiling of Col × Cvi and Cvi × Col seed embryo and endosperm showed that the abundance of these sRNAs scales with the Cvi allele dosage, strongly suggesting that this sRNA population is generated from the three *VIM* loci in a Cvi-specific and dose-dependent manner (Fig. 1*C* and *SI Appendix,* Fig. S1 *A*-*C*).

Based on ecotype-specific genomic structural differences reported in previous whole-genome comparison studies (6, 19), we further validated our findings using the chromosome-level de novo assembled reference genome of Cvi, Cvi2.0. This analysis confirmed the presence of a distinct sRNA population exclusive to Cvi allele-containing samples at loci corresponding to Cvi’s orthologs of *VIM4, VIM2,* and *VIM3,* namely, *VIM4_Cvi_* (ATCVI-1G79960 for AT1G66040), *VIM2_Cvi_* (ATCVI-1G79970 for AT1G66050), and *VIM3_Cvi_* ATCVI-5G57450 (AT5G39550), respectively (Fig. 1 *B* and *C* and *SI Appendix,* Fig. S1*D* and Dataset S2). Notably, no significant sRNA production was observed from the genomic loci encoding the other three *VIM* family genes, *VIM1* (AT1G57820/ATCVI-1G72060), *VIM5* (AT1G57800/ATCVI-1G72050), and *VIM6* (AT4G0 8590/ATCVI-4G29100), in any of the sRNA-Seq datasets analyzed (*SI Appendix,* Fig. S2). These results indicated that the first exon regions of *VIM2*, *VIM3,* and *VIM4* in Cvi2.0 genome are likely the genomic origins of the Cvi-specific sRNA population.

Although the first exons of *VIM2_Col_* and *VIM4_Col_* share extremely high sequence similarity, they are distinguishable by several SNPs. In contrast, the first exons (i.e. coding sequences) of *VIM2_Cvi_* and *VIM4_Cvi_* are 100% identical at the sequence level (*SI Appendix,* Fig. S3*A*). Meanwhile, the sequence similarity between the first exon of *VIM4_Cvi_* /*VIM2_Cvi_* and *VIM3_Cvi_* was found to be 94.1% (*SI Appendix,* Fig. S3*A*), allowing us to distinguish sRNA reads uniquely aligning to each locus. To do so, we first retrieved all sRNAs aligned to *VIM4_Cvi_*, *VIM2_Cvi_*, and *VIM3_Cvi_* from Cvi2.0-aligned sRNA-seq reads, and then realigned them to the cDNA sequences of *VIM4_Cvi_* and *VIM3_Cvi_*, respectively (*SI Appendix,* Fig. S3*B*). Of the total 253,123 sRNA reads, 30.8% mapped to both cDNA sequences. Among the remaining 69.2% of reads, the vast majority (174,844 reads, 99.7%) uniquely aligned to the *VIM4_Cvi_* cDNA sequence, with most mapping to the first exon regions (*SI Appendix,* Fig. S3 *C* and *D*). In contrast, only 354 reads (0.14%) uniquely aligned to *VIM3_Cvi_*, and these were primarily located around the first exon region that shares the same sequences as *VIM4_Cvi_*. Furthermore, uniquely aligned sRNA reads to the *VIM4_Cvi_* first exon were found specifically in regions containing SNPs unique to *VIM4_Cvi_* (and *VIM2_Cvi_*, *SI Appendix,* Fig. S3*D*). Together, these results strongly suggested that the genomic origin of this sRNAs population is the first exon region of *VIM4_Cvi_*and/or *VIM2_Cvi,_* rather than *VIM3_Cvi_*.

Three additional genomic loci were identified as producing sRNAs in a Cvi allele-dependent manner (*SI Appendix,* Fig. S4 *A*–*C* and Dataset S1 and S2). Further analysis using the Cvi2.0 genome revealed that one of these sRNA cluster loci spans two *PAI* family genes, *PAI1_Cvi_* and *PAI4_Cvi_* (Fig. 1*D* and Dataset S3), while the other two loci correspond to the *PAI2_Cvi_* and *PAI3_Cvi_* genes, respectively (*SI Appendix,* Fig. S4*D* and Dataset S2). Several *Arabidopsis* ecotypes, including Ws and Cvi (15, 16), have been reported to carry increased copy numbers of *PAI* gene family members due to an inverted duplication of the *PAI1_Cvi_* on chromosome 1, resulting in formation of a *PAI4_Cvi_*–*PAI1_Cvi_* IR. Previous studies in the Ws ecotype demonstrated that this *PAI4_Cvi_*–*PAI1_Cvi_* IR structure acts as a causal variant that triggers epigenetic silencing of endogenous homologs through non-CG methylation, mediated by an RNA-directed DNA methylation (RdDM) and CHROMOMETHYLASE3 (CMT3)/ SU(var)3-9 homologue 5 (SUVH5)-mediated pathway (15, 20–22). This mechanism aligns well with our Cvi-specific observation of abundant sRNA production from *PAI*-encoding loci. In contrast, no prior reports or evidence have suggested sRNA production from the *VIM* genes. This prompted us to further investigate this previously unreported production of sRNA at the *VIM* family locus in the Cvi genome.

### A Cvi-Specific 2.7-kb Deletion Between *VIM2* and *VIM4* Forms a Shortened IR Which Is Linked to sRNA Production.

**Fig. 2.**
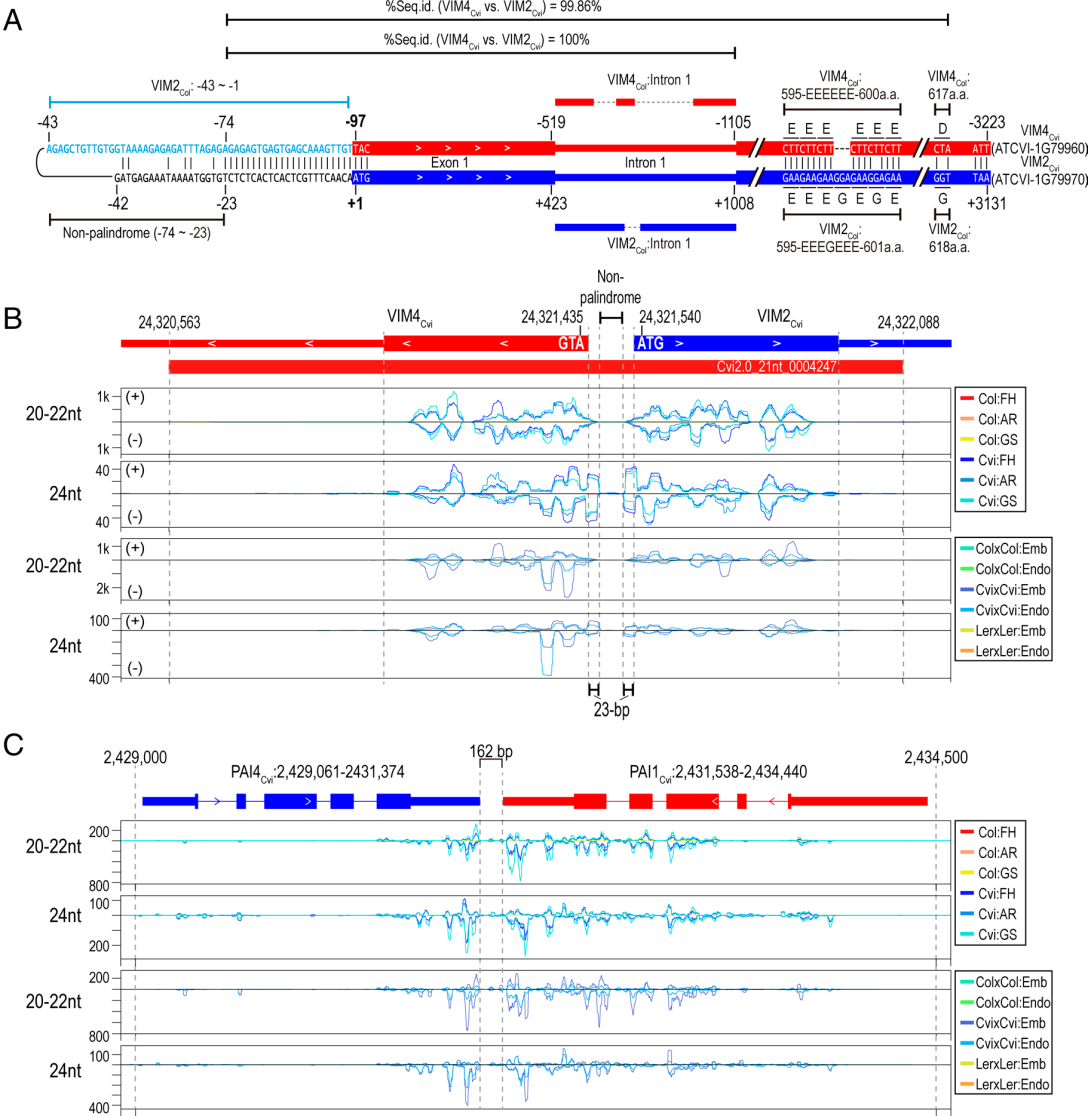
Short intergenic region-mediated palindrome structure between two neighboring *VIM* genes is causal for sRNA production in the Cvi ecotype. (*A*) Stem-loop-like presentation of a palindrome structure between ATCVI-1G79960 and ATCVI-1G79970. Sequences colored in sky blue represent *VIM2_Col_*’s 5′ UTR-homologous ATCVI-1G79960’s upstream sequences. Sequence identity % between ATCVI-1G79960 and ATCVI-1G79970 are indicated. Relative positions of 1st intron sequences of *VIM2_Col_* and *VIM4_Col_* conserved in corresponding homologs in Cvi are represented above and below the palindrome structure. (*B* and *C*) Read distribution of sRNAs across a palindrome region between ATCVI-1G79960 and ATCVI-1G79970 (*B*) and between *PAI4_Cvi_* and *PAI1_Cvi_* (*C*). A small RNA cluster identified in this region is represented as a red rectangle below gene models of two Cvi genes. The height of peaks represented in tracks indicates CP10M-normalized sRNA read coverage. FH; freshly harvested, AR; after-ripening GS; germination-stimulated, Emb; embryo, Endo; endosperm.

Revisiting the sRNA-Seq dataset using the Cvi2.0 genome and comparing the results with those from TAIR10 revealed that the intergenic region between *VIM4_Cvi_* and *VIM2_Cvi_* is substantially shorter in Cvi2.0 (~96 bp between their start codons) than in TAIR10 (~2.8-kb) (Fig. 1*B*). Additionally, sequence-level comparison of the two intergenic regions revealed that a 55-nt sequence upstream of the *VIM4_Cvi_* start codon perfectly matches the 5′ UTR of *VIM2_Col_* (Fig. 2*A*, shown in sky blue). Combined with the presence of PacBio long reads and short WGS reads derived from Cvi that fully or partially spanned this region (Dataset S4) (6), these findings suggest that both an inversion and a large deletion occurred in this region in the Cvi2.0 genome. Although the exact boundaries of the inversion could not be precisely defined, we found that the “EEEEEE”- encoding the CDS region of *VIM4_Cvi_* and “EEEGEEE”- encoding the CDS region of *VIM2_Cvi,_* are identical to the corresponding CDS region of *VIM4_Col_* and *VIM2_Col_*, respectively (Fig. 2*A*). This observation supports the possibility that the inversion breakpoints lie within the genic regions of *VIM4_Cvi_* and *VIM2_Cvi_*.

The sequence identity between *VIM4_Cvi_* and *VIM2_Cvi_* from start to termination codon was 99.86% (Fig. 2*A*), which is even higher than that between *VIM2_Col_* and *VIM4_Col_* (92.22%). This elevated similarity between *VIM4_Cvi_* and *VIM2_Cvi_*is largely due to the presence of a 100%-identical first intron sequence in both genes, which are also longer than those of *VIM4_Col_* and *VIM2_Col_* (Fig. 2*A*). In both the Cvi2.0 and TAIR10 genomes, *VIM4* and *VIM2* are arranged in a head-to-head orientation. In Cvi, the large intergenic region deletion results in the formation of a ~6.4-kb IR structure, leaving a nonpalindromic 50-bp segment in the middle (Fig. 2*A*, “−74 ~ −23”). Notably, since *VIM4_Cvi_* and *VIM2_Cvi_* showed 100% sequence identity across their first exon and first intron, this *VIM4*–*VIM2* IR could potentially form a double-stranded RNA (dsRNA) stem-loop structure, serving as a substrate for sRNA biogenesis, similar to the known *PAI4*–*PAI1* IR) structure (Fig. 2*C*).

Due to the lack of publicly available DCL-knockout lines in the Cvi background, we took an alternative approach to assess the potential involvement of DCLs in sRNA production within the *VIM4_Cvi_*–*VIM2_Cvi_* and *PAI4_Cvi_*–*PAI1_Cvi_* regions by estimating phasing scores (*SI Appendix*, Fig. S5). ShortStack-measured phase scores exceeded the defined threshold, suggesting DCL activity in sRNA production from these regions (Dataset S2). To further examine this, we calculated phase score using sRNAs mapped to these loci and detected phased signals in both regions (*SI Appendix*, Fig. S5 *A* and *B*). However, these patterns were less distinct than those observed at *AtTAS1B* (*SI Appendix,* Fig. S5*C*), a well-characterized trans-acting siRNA (tasiRNA) generating locus. This indicates that sRNA production from the *VIM4_Cvi_*–*VIM2_Cvi_* and *PAI4_Cvi_*–*PAI1_Cvi_* regions is unlikely to follow a canonical phasing mechanism. Instead, the observed patterns may reflect the processive action of a specific DCL isoform, whereas their lower distinctness compared to *AtTAS1B* may suggest the combined activity of multiple DCL isoforms on a single substrate.

Consistent with this potential dsRNA-generating IR structure, sRNAs reads were enriched across the stem-forming regions: first exon and its upstream 23-bp sequences of *VIM4_Cvi_* and *VIM2_Cvi_* (Fig. 2 *A* and *B*), whereas relatively few sRNA reads mapped to the loop-forming sequence (Fig. 2*B*). This sRNA distribution pattern closely resembled those previously reported for the *PAI4*–*PAI1* IR and the transgene encoding *Block C* IR (*SI Appendix,* Fig. S6) (23). In contrast, no significant structural variation was identified at the *VIM3_Cvi_* locus to explain the first exon-enriched sRNA distribution. Overall, these results suggest that the Cvi-specific *VIM4_Cvi_*–*VIM2_Cvi_* arrangement represents a causal structural variant underlying Cvi allele-dependent sRNA production.

### 5′ End-Extended, Intergenic Region-Spanning Alternative Transcripts of *VIM2_Cvi_*.

**Fig. 3.**
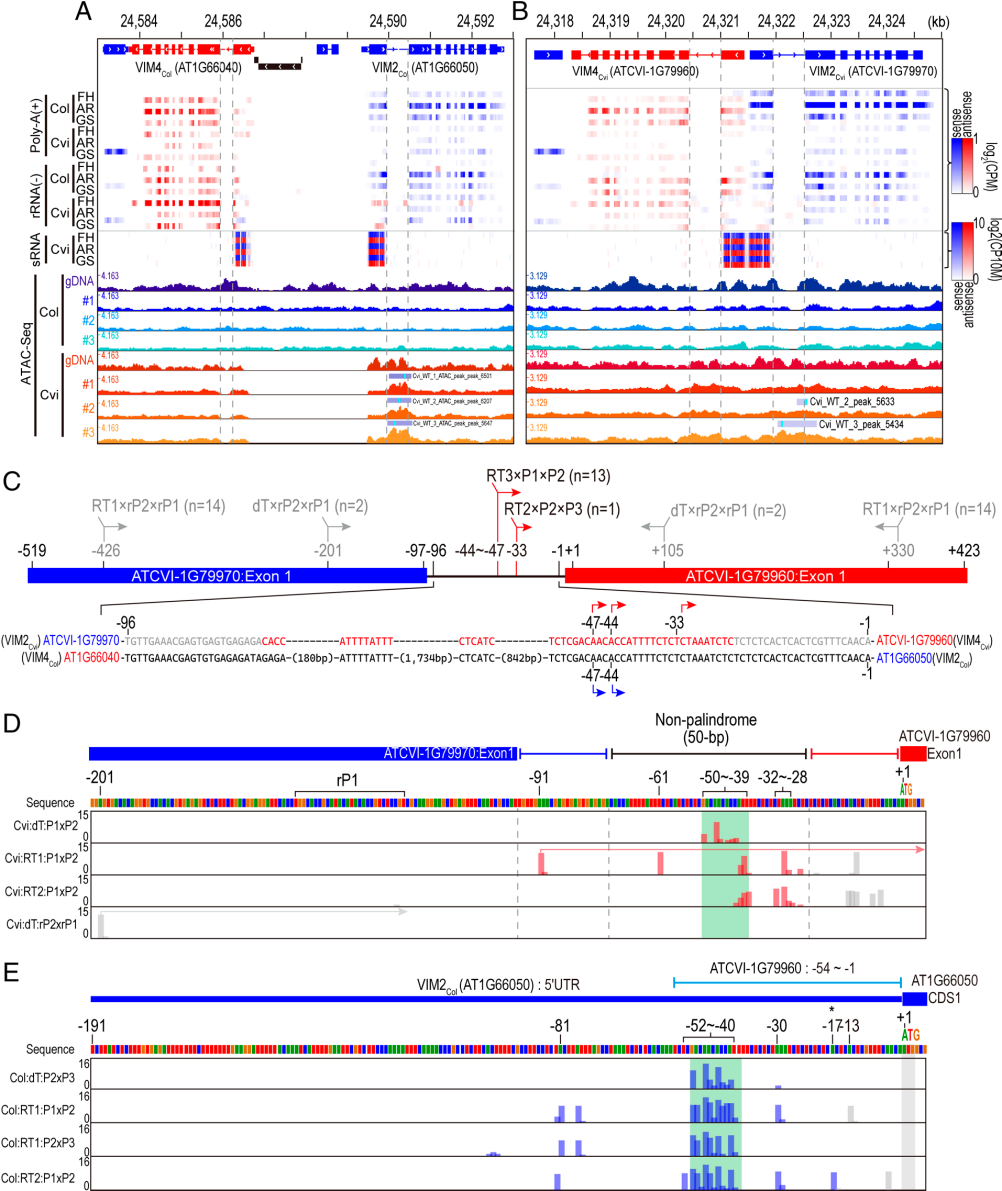
Alternative transcription start sites matching the gene orientation of ATCVI-1G79960 are located in the first exon of ATCVI-1G79970. (*A* and *B*) Distribution of transcriptome, sRNA-Seq, and ATAC-Seq datasets across a palindrome region between *VIM2* and *VIM4* on TAIR10 (*Left* panel) and Cvi2.0 (*Right* panel). “PolyA(+)” and “rRNA(–)” on the left of tracks represent poly-A-selected and rRNA-depleted transcriptome datasets, respectively. The height of tracks corresponding to the ATAC-Seq dataset indicates CPM-normalized base coverage. Gray, dotted lines represent the 1st intron region of *VIM* genes. ATAC-Seq peaks defined by MACS are represented by boxes with labels on each track. (*C*) Schematic representation of the 5′ end position and orientation of sequenced tags identified by a classic 5′ RACE approach. Label on each identified position represents primers (separated by a cross mark) used in reverse transcription (“RT#”), 1st-round PCR and 2nd-round PCR, respectively. Positions and arrows colored in gray represent the 5′ end of sequenced tags whose origin is uncertain due to the perfect sequence homology between two *VIM* genes in the palindrome, while red- and blue-colored positions/arrows represent the 5′ end of sequenced tags whose orientation matches the gene orientation of ATCVI-1G79960 and AT1G66050 (*VIM2_Col_*), respectively. (*D* and *E*) 5′ end position of sequenced tags identified by targeted 5′ RACE-Seq. The peak height represents log_2_-transformed raw number of tags having same 5′ end position, and peak color represent the sequenced tags whose orientation matches the gene orientation of ATCVI-1G79960 (colored in red) or AT1G66050 (*VIM2_Col_*, colored in blue), respectively. Label on the left of tracks represents primers (separated by a cross mark) used in reverse transcription (“RT#”), 1st-round PCR and 2nd-round PCR, respectively. Green boxes indicate the region considered as the major transcription start site of ATCVI-1G79960 (“−50 ~ −39”) or AT1G66050 (*VIM2_Col_*, “−52 ~ −40”).

Because the sequence separating the *VIM4_Cvi_*–*VIM2_Cvi_* IR is only 50 bp—shorter than the 5′ UTRs of *VIM4_Col_* (79 nt) and *VIM2_Col_* (191 nt), we hypothesized that transcription of *VIM4_Cvi_* and *VIM2_Cvi_* might start within the genic region and extend across the intervening IR sequence. To test this, we analyzed strand-specific RNA-Seq datasets generated from both poly (A)-selected and rRNA-depleted RNA samples. However, neither dataset revealed transcripts spanning the IR-intervening sequence in the Cvi genome. Instead, several reads aligned to the antisense strand of the first exons of *VIM4_Cvi_* and *VIM2_Cvi_* in the rRNA-depleted transcriptomes (Fig. 3 *A* and *B*), while sense-strand reads were barely detected in the same exon regions, where sRNAs were predominantly produced (Fig. 3*A*). The presence of antisense-specific reads suggests the existence of transcription start sites (TSSs) within this IR region. Supporting this, public ATAC-seq data from Cvi-0 (24) revealed open chromatin across the *VIM4*–*VIM2 IR* region (Fig. 3 *A* and *B*).

To determine the TSSs and transcribed regions of *VIM2_Cvi_* and *VIM4_Cvi_*, 5′ RACE was conducted using RNAs extracted from after-ripening (AR) stage seeds of Cvi and Col (*SI Appendix,* Fig. S8). This analysis identified several 5’ RACE tags within the 50-bp intergenic region between *VIM4_Cvi_* and *VIM2_Cvi_*, supporting the presence of *VIM4_Cvi_* TSSs (Fig. 3*C*). Notably, two of the three *VIM4_Cvi_* TSSs (“−44” and “−47” in Fig. 3*C*) precisely matched the experimentally validated TSSs of *VIM2_Col_* (Fig. 3*C* and *SI Appendix,* Fig. S9*A*), suggesting that *VIM4_Cvi_* and *VIM2_Col_* share similar TSS positions. Furthermore, we detected 5′ RACE tags—exclusively in Cvi seed samples —mapping to the antisense strand of the first exon of *VIM4_Cvi_* and *VIM2_Cvi_* (Fig. 3*C*), suggesting transcription in the opposite direction to *VIM4_Cvi_* or *VIM2_Cvi_*.

The identical sequences of *VIM4_Cvi_* and *VIM2_Cvi_* first exons made it unclear from where antisense transcription originated. Moreover, the hypothesized stem-loop structure from the above results (Fig. 2) could not be verified due to the lack of 5′ RACE tags spanning the intergenic region. To address this, we performed high-throughput sequencing of the amplified 5′ RACE tags with Illumina MiniSeq. In the 5′ RACE-Seq data from Cvi, we identified numerous 5′ RACE tags with 5′ ends mapped between −50 and −39-bp upstream of the *VIM4_Cvi_* start codon (Fig. 3*C*). Similarly, in Col samples, 5′ RACE tags were detected within a comparable upstream region, between −52 and −40-bp from the *VIM2_Col_* start codon (Fig. 3*D*). The consistent enrichment of 5′ RACE tags at nearly identical distances upstream of both *VIM2_Col_* and *VIM4_Cvi_* suggests that this region serves as the major TSS for *VIM4_Cvi_*in Cvi 2.0 genome.

In addition to identifying the major TSS of *VIM4_Cvi_*, we also detected 5′ RACE tags that span the intergenic region, with 5′ ends located 91-bp upstream of the *VIM4_Cvi_* start codon. Furthermore, 5′ RACE-Seq revealed additional putative TSSs on the antisense strand within the first exon region of *VIM4_Cvi_* /*VIM2_Cvi_*, specifically at positions −201 and +105 (*SI Appendix,* Fig. S9*E*). While several 5’ RACE tags aligned in the same direction as *VIM4_Cvi_*transcription, only a few reads in the *VIM2_Cvi_* transcription direction were found, located 30-bp upstream of the *VIM2_Cvi_* start codon only (*SI Appendix,* Fig. S9*E*). This strand-specific bias in 5′ RACE tag distribution—favoring *VIM4_Cvi_* transcription across the intergenic palindrome—strongly suggests that *VIM4_Cvi_* transcription predominantly occurs across this palindrome region in Cvi. Furthermore, the identification of alternative *VIM4_Cvi_* TSSs beyond the nonpalindrome segment, including the first *VIM2_Cvi_* exon, suggests that *VIM4_Cvi_* may produce 5’ UTR-extended transcripts. These transcripts could potentially form stem-loop structures for sRNA biogenesis.

### *VIM4_Cvi_*–*VIM2_Cvi_* IR-Derived sRNAs Negatively Regulate *VIM* Family Genes in Trans and Induce RdDM in cis.

**Fig. 4.**
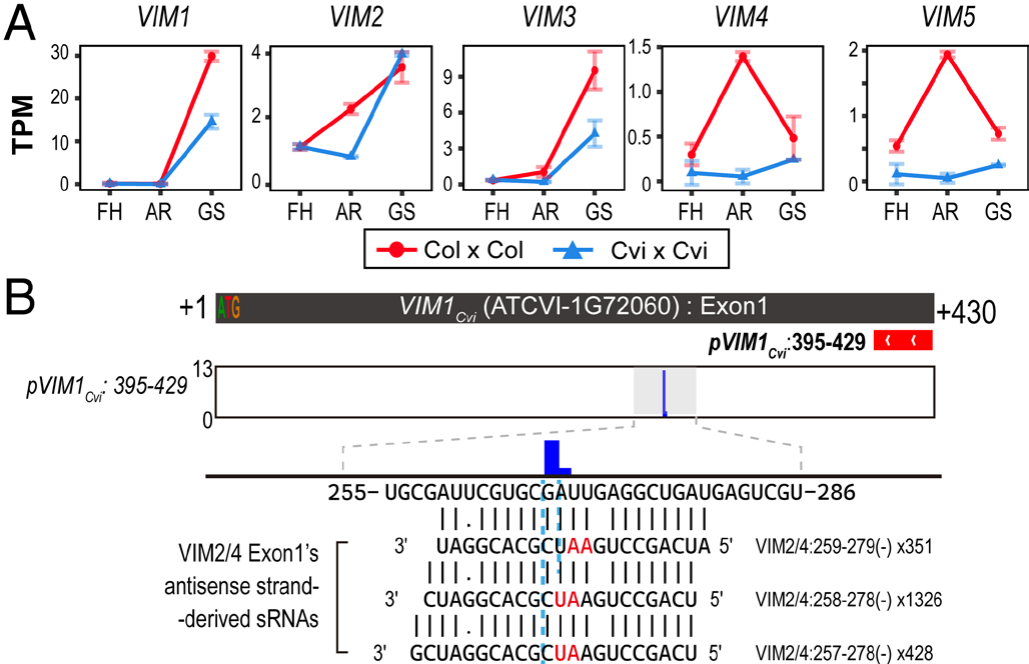
*VIM4_Cvi_*–*VIM2_Cvi_* IR-derived sRNA negatively regulates *VIM1* through posttranscriptional silencing. (*A*) TPM-normalized mean expression levels of five *VIM* family genes measured from poly(A)-selected transcriptome datasets that are produced from Col (Col × Col, red) and Cvi (Cvi × Cvi, blue). FH; freshly harvested, AR; after-ripening, GS; germination-stimulated. (*B*) 5′ end of sequence tags identified by the target 5′ RACE-Seq against *VIM1_Cvi_* transcript. The red box represents a primer for 2nd-round PCR. Peak height represents the log_2_-transformed raw number of tags sharing a 5′ end position. Target sequences in the *VIM1_Cvi_* transcript and corresponding *VIM4_Cvi_*–*VIM2_Cvi_* IR-derived sRNA sequences are represented beneath. *VIM4_Cvi_*–*VIM2_Cvi_* IR-derived sRNA sequence labels indicate origin relative to start codon in first exon of *VIM2/4*, numbers indicate raw sRNA reads detected from all Cvi seed sRNA-Seq datasets. Red sequences represent 10th and 11th position of *VIM4_Cvi_*–*VIM2_Cvi_* IR-derived sRNAs, respectively, and predicted cleavage sites that match the identified 5′ RACE tags are indicated by sky blue dotted lines.

Strand-specific RNA-Seq analysis from developing seeds of Col and Cvi revealed that expression levels of the five *VIM* family genes were lower in Cvi than in Col at least at one of three developmental stages examined (Fig. 4*A*). This expression pattern is consistent with a previous observation in endosperm from Col × Col vs. Col × Cvi crosses (*SI Appendix,* Fig. S10) (25). Given the abundant production of sRNAs from *VIM2, VIM3,* and *VIM4* loci, and the high sequence similarity among *VIM* family members, it is possible that these sRNAs could target *VIM* family gene transcriptions both *in cis* and/or *in trans*.

To examine this hypothesis, 5′ RACE-Seq was performed to detect cleaved transcripts carrying a monophosphate at the 5′ end, a hallmark of sRNA-mediated cleavage. This analysis identified a distinct cleaved tag within the first exon of *VIM1_Cvi_*, corresponding to the predicted cleavage site of 21-/22-nt sRNAs derived from *VIM2/3/4*, but only when *VIM1*-specific primers were used (Fig. 4*B*). No cleavage tags were detected when using *VIM2/3/4/5_Cvi_*-specific primers. Combined with the reduced *VIM1* transcript levels observed in Cvi transcriptome these results support the idea that *VIM4_Cvi_*–*VIM2_Cvi_* IR-derived sRNAs can act *in trans* to target *VIM1_Cvi_* through a sRNA-mediated posttranscriptional silencing pathway.

**Fig. 5.**
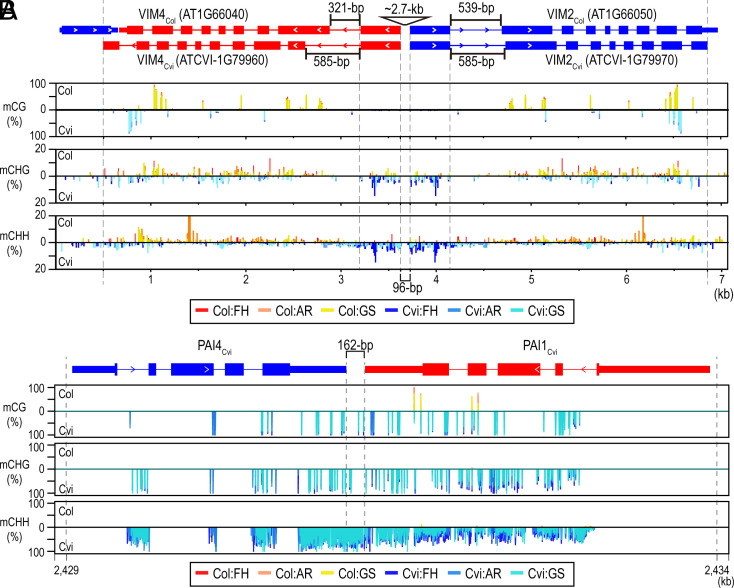
Non-CG DNA methylation contexts at two sRNA-producing palindrome regions are hypermethylated in Cvi. (*A*) DNA methylation level distribution on three cytosine methylation contexts across the *VIM4*–*VIM2* palindrome region. Because of the structural variation across in this region between Col and Cvi, ~2.7-kb of intergenic sequences between *VIM2_Col_* and *VIM4_Col_* in TAIR10 were removed to synchronize the 1^st^ exon’s coordinates of those to that of *VIM2_Cvi_* and *VIM4_Cvi_*, respectively. After that, whole-genome bisulfite sequencing (WGBS) reads from Col’s seed samples were mapped to this intergenic region-removed ~7-kb sequence fragment to estimate a DNA methylation level. Cvi’s WGBS reads were mapped to a ~7-kb sequence fragment including *VIM2_Cvi_* and *VIM4_Cvi_*. The height of bar graph represents DNA methylation level measured from WGBS datasets. (*B*) DNA methylation level distribution on three cytosine methylation contexts across the *PAI4*–*PAI1* palindrome region. Represented DNA methylation levels of seed samples from Col and Cvi were measured after WGBS read alignment to the Cvi2.0 reference sequence.

IR-derived sRNAs are known to induce cytosine methylation at their target genomic sites through the RdDM pathway (23, 26). To assess whether the *VIM4_Cvi_*–*VIM2_Cvi_* IR-derived sRNAs influence RdDM-mediated non-CG DNA methylation, we analyzed whole-genome bisulfite sequencing (WGBS) datasets from our previous study (5). This analysis revealed that the first exon regions of *VIM2_Cvi_* and *VIM4_Cvi_* exhibited increased DNA methylation in both non-CG methylation contexts (CHG and CHH) compared to Col (Fig. 5*A*). Similarly, the first exon of *VIM3_Cvi_* also exhibited elevated non-CG methylation levels compared to Col (*SI Appendix,* Fig. S11*A*), whereas *VIM1* showed no significant differences in DNA methylation across any cytosine context between Col and Cvi (*SI Appendix,* Fig. S11*B*). We also confirmed increased non-CG methylation across the *PAI4_Cvi_*–*PAI1_Cvi_* IR region in Cvi based on WGBS data (Fig. 5*B*), consistent with previous observations in the Ws strain (16, 22, 27). Notably, while the *PAI4_Cvi_*–*PAI1_Cvi_* IR exhibited strong hypermethylation in both CG and non-CG contexts, the *VIM4_Cvi_*–*VIM2_Cvi_* IR showed comparatively weaker hypermethylation. These results suggest that sRNAs derived from the *VIM4_Cvi_*–*VIM2_Cvi_* IR can contribute to non-CG methylation both *in cis* and *in trans*, but this effect appears to be limited to the *VIM2/3/4* genes and is relatively modest compared to the methylation induced by the *PAI4_Cvi_*–*PAI1_Cvi_* IR.

### The *VIM4–VIM2* IR Locus Is Highly Polymorphic Across *A. thaliana* Populations and Is Conserved at a Genus-Level.

While the *PAI4*–*PAI1* IR has been reported in several Arabidopsis ecotypes (16), the *VIM4–VIM2* IR and structural variation in this genomic region have not been previously characterized. To identify other ecotypes that possess a Cvi-like shortened intergenic region, we analyzed synteny at the *VIM4–VIM2* IR locus using chromosome-level genome assemblies from 96 accessions [69 from Lian et al. (19), and 27 from Kang et al. (28)]. However, none of the accessions we analyzed had the highly shortened *VIM4–VIM2* intergenic region (*SI Appendix,* Fig. S12), suggesting that the Cvi-like IR structure is a very rare, if not unique, genomic variant among *A. thaliana*’s accessions.

**Fig. 6.**
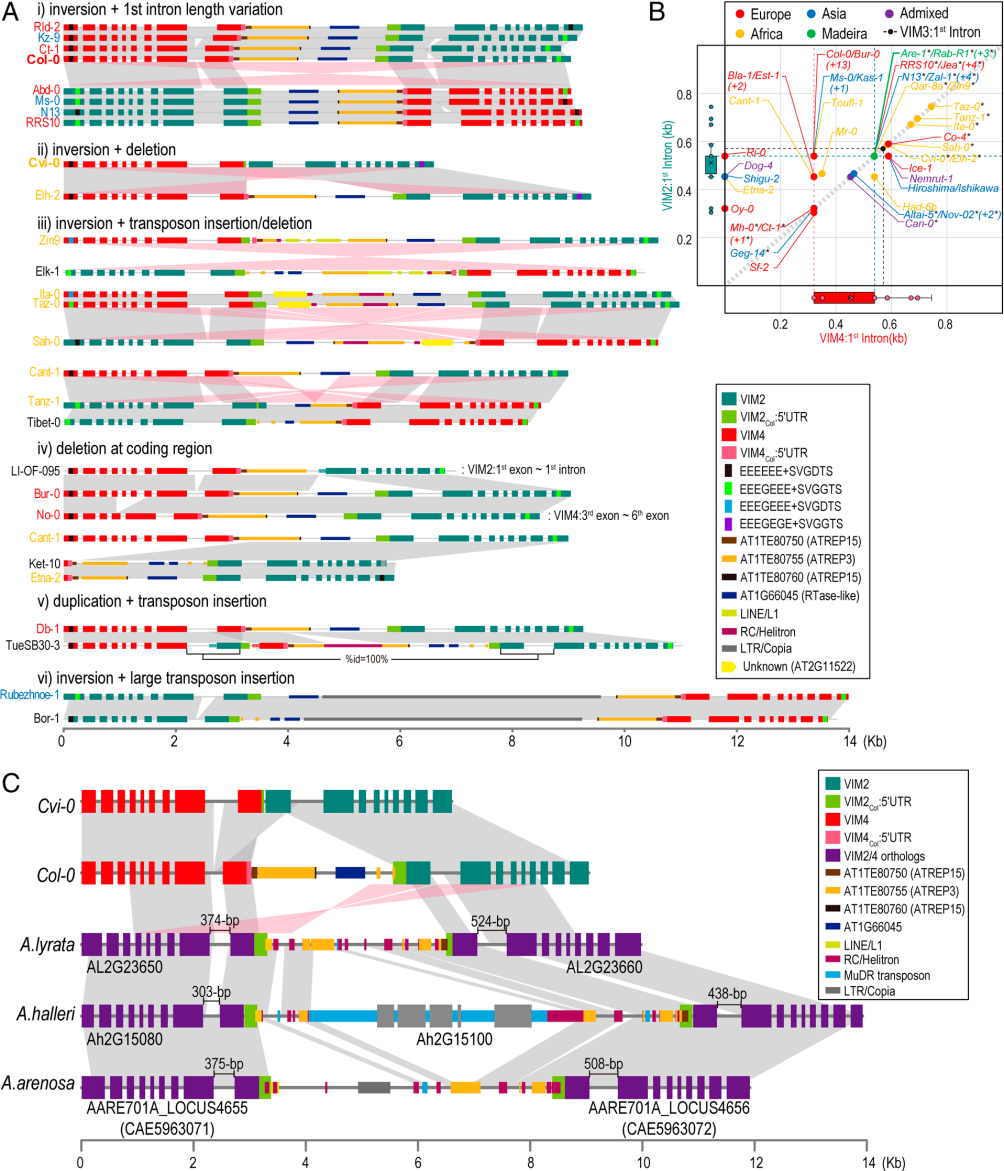
The *VIM4*–*VIM2* IR region is highly polymorphic across *A. thaliana* ecotypes and is conserved in plants of the genus *Arabidopsis*. (*A*) Schematic diagram of structural variation in a subset of the 96 accessions of *A. thaliana*. Exons of *VIM2* and *VIM4* annotated by Lian et al. are represented by turquoise green and pink, respectively. 5′ UTR sequences of *VIM2_Col_* and *VIM4_Col_* are represented by light green and red, respectively. Colored boxes located between *VIM2* and *VIM4* represent sequences encoding transposable elements and those homologous to gene fragments. The gray or pale-red links between the boxes represent homologous relationships analyzed by *nucmer* in MUMmer package (see Methods). Colors of accession names on the left represent the genetic classification of each accession provided by Lian et al (19), which is “Europe” (red), “Madeira” (green), “Asia” (blue), “Africa” (orange), and “Admixed” (violet). The range written to the right of the *VIM4*–*VIM2* IR shown in “iv)” (LI-OF-095 and No-0) indicates the region where the deletion occurs. (*B*) Pairwise distribution of the 1st intron length of *VIM2* and *VIM4* homologs among 69 *A. thaliana* accessions analyzed by Lian et al. (19). Accessions labeled with a black asterisk have perfectly symmetric 1st introns at the nucleotide level. Colors of dots and accession names represent the genetic groups of accessions that were categorized by Lian et al. (19). The green dashed line represents the most frequently observed 1st intron length of *VIM2* (539-bp, 47 of 69 accessions), whereas the pink and blue dashed lines represent the most (321-bp, 27 of 69 accessions) and the second-most frequently observed (539-bp, 20 of 69 accessions) 1st intron length of *VIM4*, respectively. (*C*) *VIM4*–*VIM2* IR region is conserved in *A. lyrata*, *A. halleri,* and *A. arenosa*. Gene structures of *VIM2/3/4* family homologs in *A. lyrata*, *A. halleri,* and *A. arenosa* are represented by violet rectangles. The gray or pale-red links between the boxes represent homologous relationships analyzed by nucmer in MUMmer package (default option).

However, we did find extensive structural polymorphism at this locus, including insertions, deletions, duplications, and inversions (Fig. 6*A* and *SI Appendix,* Fig. S12 *A* and S11 *B* Flanking boundaries of *VIM4*–*VIM2* IR are highly conserved (*SI Appendix,* Fig. S13), while C-terminal coding regions at *VIM2*/*VIM4* are inverted in certain accessions (e.g., Col-0/Kz-9 vs. Ms-0; Fig. 6*A*). Together, these findings indicate that inversions between *VIM2* and *VIM4* frequently occur within the IR, although the precise breakpoint may vary, likely due to the near-perfect sequence homology across this region.

Since many structural variations are located near the first intron of *VIM2* and/or *VIM4*, we further analyzed the first intron of various accessions. This analysis revealed that intron length is primarily driven by tandem repeats copy number, each 75 to 83-nt long. (*SI Appendix,* Fig. S13). Together with the presence of the *VIM4–VIM2* IR structure, these intronic repetitive sequences likely promote homologous recombination, both within individual introns and between the first introns of *VIM2* and *VIM4*, thereby contributing to the extensive polymorphism observed in the *VIM4*–*VIM2* IR region.

Unlike the limited distribution of the *PAI4*–*PAI1* IR across *A. thaliana* ecotypes (16), the *VIM4*–*VIM2* IR is broadly present among most *A. thaliana* accessions, suggesting that it may have been inherited from a common ancestor. To explore the origin and conservation of this *VIM4*–*VIM2* IR genotype, we identified homologs of the *A. thaliana*’s six *VIM* family genes using BLASTP (<1E–100) against protein sequences from 35 *Brassicaceae* and 3 non-*Brassicaceae* species. This analysis identified 174 homologous proteins across these species (*SI Appendix,* Fig. S15 and Dataset S6). Further comparative genomic analysis revealed that an IR structure containing two highly homologous *VIM2/3/4* family genes is present in three close relatives of *A. thaliana* (Fig. 6*C*). In contrast, other related genera, such as *Capsella*, *Malcolmia,* and *Boechera* (lineage “Camelinodae” in *SI Appendix,* Fig. S15), possess only one *VIM2/3/4* family homolog. Notably, the IR structures observed in the three other *Arabidopsis* species also harbor Class II-type TEs homologous to those found between *VIM4* and *VIM2* in *A. thaliana*. In addition, the first intron lengths of neighboring *VIM2/3/4* homologs are asymmetric in all three *Arabidopsis* species, indicating a pattern of sequence variation similar to that seen across *A. thaliana* ecotypes.

Taken together, these findings suggest that the *VIM2/3/4* IR structure in the genus *Arabidopsis* likely originated from an ancient intrachromosomal inverted duplication event that occurred prior to the divergence of *A. thaliana*. This also implies that the Cvi-specific *VIM4*–*VIM2* IR genotype, along with other rare genotypes, is an evolutionary outcome of lineage-specific structural variation, rather than a remnant of the ancestral form.

## Discussion

Here, we demonstrate that natural genetic variation at the *VIM4_Cvi_*–*VIM2_Cvi_* IR locus alters posttranscriptional gene silencing of *VIM* family genes via siRNAs derived from the *VIM4_Cvi_*–*VIM2_Cvi_* IR (Fig. 4). In turn, this likely reduces genome-wide CG methylation via the VIM1-3–MET1 pathway; i.e. a reduction in VIM1-3-mediated MET1 access to DNA, and subsequent reduction in mCG catalysis. This scenario is consistent with the phenotype of previously published genetic knockdowns of *VIM1-3* in Col-0, where CG methylation is reduced genome-wide (9).

Recent findings by Briffa et al. indicate that genic mCG patterns are shaped by genetic factors modulating de novo methylation and maintenance, yet remain dynamic through epigenetic fluctuations occurring in a millenia-timescale that can last thousands of years (29). Their mathematical model successfully predicts the overall mCG patterns on genic region for almost accessions of *A. thaliana* by modulating two model parameters—cooperative interaction strength (r^*^) and de novo activity level ($r_{0}^{+}$). Interestingly, reproducing the overall gbM patterns of Cvi-0 and other low-methylation accessions through their model required a simultaneous reduction in both parameters. This suggests that these accessions have gradually lost mCG level and occupancy across the genome, likely due to an attenuated activity in mCG establishment and maintenance driven by specific genetic changes. Since the VIM gene family plays a vital role in establishing and maintaining mCG methylation across the plant genome, the suppression of *VIM1* by *VIM4_Cvi_*–*VIM2_Cvi_* IR-derived sRNAs offers a plausible genetic mechanism for the reduced DNA methylation maintenance in Cvi-0. Considering that Cvi-0 has evolved in geographical isolation for approximately 5,000 years (2), this sRNA-mediated pathway, triggered by structural variation, likely drove a long-term shift in the methylome’s steady state, effectively recapitulating the millennial-timescale fluctuations predicted by the model of Briffa et al. (29). For Can-0 and UKID116, it is likely that yet-to-be-identified genetic variations are responsible for their reduced global mCG levels, providing a broader context for the genetically determined epigenetic states observed in these accessions.

Our multiaccession, sequence-level comparisons of the *VIM4*–*VIM2* locus revealed that the regulatory circuit between *VIM* family genes and *VIM4_Cvi_*–*VIM2_Cvi_* IR-derived siRNAs likely originated from natural sequence variation at this locus. This unique genotype appears to have undergone multiple modes of genome rearrangement, acting on a number of repetitive regions, from short tandem repeats within the first intron (Fig. 6*B* and *SI Appendix,* Fig. S14) to a long palindromic structure formed between two highly homologous genes (Fig. 6 and *SI Appendix,* Fig. S12). These complex rearrangements likely contribute to the high degree of polymorphism at the *VIM4*–*VIM2* locus across *A. thaliana* populations. Interestingly, ecotypes that are geographically isolated—such as those in the CVI and “Madeira” groups (Are-1, Are-2, Are-6, Are-10, and Rab-R1)—tend to exhibit a fixed *VIM4–VIM2* genotype (*SI Appendix,* Fig. S12), suggesting local fixation of structural variants. Intriguingly, Can-0 is also geographically isolated from continental populations and is also CG hypomethylated. However, its *VIM4*–*VIM2* genotype is similar to those in accessions distributed across Asia (e.g., Kar-1, Kz-9, Hiroshima) or Europe, unlikely to generate siRNA in a manner analogous to Cvi (*SI Appendix,* Fig. S12), whereby a different mechanism is likely to contribute to hypomethylation in this accession.

Previously, genetic variation in the form of SNPs in genes involved in DNA methylation pathways have been linked to DNA methylation changes, using mixed model genome-wide association (GWA) analyses. Using variation in global gbM levels of *A.thaliana* accessions as the phenotype, SNPs were identified in genes encoding histone variant H2A.Z that led to decreased H2A.Z expression and increased gene-body methylation, specifically in accessions from northern Sweden. The MET1–VIM axis was also implicated in this study, where *MET1* SNPs were associated with both high and low GbM CG methylation (29, 30).

In a second analysis, SNPs in *AGO9*, *AGO1,* and *NRPD1B* were associated with changes in RdDM-mediated CHH methylation, with *MBD3*, *AGO9,* and *CMT2* SNPs associated with CMT2-mediated CHH methylation (3). Our WGBS analysis revealed CHH hypermethylation at the first exons of *VIM3_Cvi_*, *VIM4_Cvi_*, and *VIM2_Cvi_* was comparable to other accessions (Fig. 5*A* and *SI Appendix,* Fig. S10*A*), suggesting that sRNAs derived from the *VIM4_Cvi_*–*VIM2_Cvi_* IR locus may mediate RdDM both *in cis* and *trans*. A similar analysis of the *PAI4_Cvi_*–*PAI1_Cvi_* IR locus also showed hypermethylation across all cytosine contexts (Fig. 5*B*). However, non-CG DNA methylation at the *VIM4_Cvi_*–*VIM2_Cvi_* was substantially lower than at the *PAI4_Cvi_*–*PAI1_Cvi_* locus. Possibly, this may reflect a lower abundance of 24-nt siRNAs relative to the 20~22-nt siRNA population at *VIM4_Cvi_*–*VIM2_Cvi_* (Fig. 1*C* and *SI Appendix,* Fig. S1), indeed the *PAI4_Cvi_*–*PAI1_Cvi_* locus appears to produce a higher proportion of 24-nt siRNAs (*SI Appendix,* Fig. S4 *A*-*C*). Alternatively, DNA methylation at *VIM4_Cvi_*–*VIM2_Cvi_* could be actively suppressed e.g. by ROS1-mediated active DNA demethylation, or antagonistic histone variants such as H2AZ (29, 30). Additionally, the establishment and maintenance of DNA methylation is known to be influenced by pre-existing methylation states, as DNA methylation clusters can self-reinforce (29). Last, given that the *PAI4*–*PAI1* IR genotype is found in Cvi as well as in several other continental accessions (16), the *VIM4_Cvi_*–*VIM2_Cvi_* locus likely emerged more recently than the *PAI4_Cvi_*–*PAI1_Cvi_* locus. As a result, insufficient evolutionary time may have elapsed for stable epigenetic variation to accumulate under fixed environmental conditions.

Our investigation into the evolutionary origin of the *VIM4*–*VIM2* IR revealed that this structure is conserved only within species of the *Arabidopsis* genus (Fig. 6*C*). Notably, we also found that *VIM3*, one of the three functionally redundant genes required for the maintenance of CG methylation in *A. thaliana* (9, 14), is present exclusively in the *A. thaliana* genome (*SI Appendix,* Fig. S15). These observations suggest *VIM3* arose via an interchromosomal duplication of *VIM2* or *VIM4* after the formation of the *VIM4*–*VIM2* IR (Fig. 6*C*) and the subsequent speciation of *A. thaliana*. Furthermore, the expansion of the *VIM2/3/4* clade through segmental duplication appears to have occurred more recently than that of the *VIM1/5/6* clade (*SI Appendix,* Fig. S15). Considering that *VIM3* likely emerged after the settlement of *VIM4-VIM2* IR and that the three redundant VIM proteins exhibit a functional hierarchy (*VIM1* > *VIM3* > *VIM2*) (9), it is plausible that the emergence of *VIM3* as the second most influential homolog represents an evolutionary adaptation. Specifically, this may have been driven by the potential dysfunction of *VIM2,* caused by the unstable genome structure of the *VIM4*–*VIM2* IR (Fig. 6*A* and *SI Appendix,* Fig. S12).

The unique CG hypomethylation of the Cvi genome makes this accession an outlier among *A.thaliana* populations (3, 4). A number of previous studies sought to understand the origins of this characteristic epigenetic profile; however, its origins remained elusive until now (3, 4, 29, 31). Here, we show that Cvi-specific structural variation at an inverted repeat between *VIM4_Cvi_* and *VIM2_Cvi_* genes results in siRNA production and posttranscriptional silencing *in trans* of the homologous *VIM1* gene. We demonstrate that this previously unrecognized regulatory layer alters endogenous expression of *VIM* family genes in Cvi. We outline the substantial structural polymorphisms within the *VIM4*–*VIM2* IR region across hundreds of *A. thaliana* ecotypes, which appear to have arisen following the formation of that IR in the *Arabidopsis* genus, likely facilitated by nonallelic homologous recombination and transposable element activity, and show that the locus structure at Cvi is unique. Together, our findings show that repeat-mediated structural polymorphisms at the Cvi-0 *VIM4*–*VIM2* IR lead to Cvi-specific RNA interference at this locus, a decrease in *VIM* gene expression, highly plausibly leading then to decreased genomic CG methylation. These observations highlight the potential of structural variants to alter epigenetic status across species.

## Materials and Methods

Please see *SI Appendix* for full details.

### Plant Materials and Growth Conditions.

Plant materials and growth conditions are described in our previous study (5). Briefly, freshly harvested (FH) seeds (fully matured green) were collected from brownish siliques that had just begun to dry in both ecotypes. After ripening (AR) seeds of Col were harvested from fully dried plants. AR seeds of Cvi were harvested from fully dried plants at a stage comparable to the Col AR stage but were further dried for an additional 60 d. Germination-stimulated (GS) seeds were prepared from AR seeds by stratification in the dark at 4 °C for three days, followed by exposure to white light for two hours and subsequent incubation in the dark at 22 °C for 24 h.

### Small RNA-Seq Analysis.

Small RNA-Seq datasets derived from FH, AR, and GS seeds of Col and Cvi from our previous study (5) are available in the NCBI SRA (Project No. PRJNA835511). Small RNA-Seq datasets derived from the embryo and endosperms of F1 seeds from reciprocal hybrids between Col, Cvi, and Ler (17) are available in the NCBI SRA (SRP099231). Small RNA-Seq datasets derived from longitudinally sampled whole seeds of Col and Cvi (18) are available in the NCBI SRA (SRP072220). Preprocessing for all small RNA datasets used in this study were performed as described previously (5).

Preprocessed sRNA reads were aligned to reference genomes using *ShortStack* (v3.8.5) with parameters (*--align_only --mismatches* 0 *--mmap* u *--bowtie_m* 1000 *--ranmax* 3) (32). For nonreference genomes, for example, Cvi and Ler, pseudogenomes were prepared by replacing the TAIR10 (Col) sequence using ecotype-specific SNPs (excluding indels) available in public databases, including TAIR and 1001GP (6). Since Cvi’s SNP information exists both in TAIR and 1001GP, all SNPs were merged and used for downstream analysis. Another reference genome sequence for Cvi (Cvi2.0) from the 1001GP database (6) was also used for aligning Cvi’s sRNA-Seq. To analyze small RNAs (sRNAs) from reciprocal F1 hybrids between two ecotypes, we constructed a pseudodiploid reference genome by concatenating the genome assemblies (two of either TAIR10, and pseudogenomes for Cvi or Ler) of the two parental ecotypes in silico. sRNA reads obtained from the hybrid were aligned to this combined reference. Following alignment using ShortStack (--bowtie_m 2000, --ranmax 6), the resulting BAM files contained chromosome IDs corresponding separately to each parental genome. To allow consistent downstream analysis including quantification, homologous chromosomes from the two parental genomes were renamed and unified under common chromosome identifiers (e.g., “chr1_Col” and “chr1_Cvi” were both renamed as “chr1”). All aligned reads were then subjected to sRNA cluster identification, normalization, quantification, and differential expression analysis as described in previous study in detail (5).

### 5′ RACE and Targeted 5′ RACE.

To confirm the 5′ ends of transcripts derived from the *VIM2* and *VIM4* loci, DNase I-treated total RNA was extracted from the seeds of Col and Cvi ecotypes. RNA samples were sequentially treated with calf intestine alkaline phosphatase (CIP, Takara) and tobacco acid pyrophosphatase (TAP, Epicentre). A 5′ RNA adapter was ligated to processed RNAs using T4 RNA ligase 1 (Ambion). Primers were designed across the first exon of ATCVI-1G79960/79970 (*SI Appendix,* Fig. S8*A* and Dataset S7) and used to synthesize cDNAs or to amplify the 5′ ends of transcripts. cDNA synthesis was performed using PrimeScript (Takara). To perform 5′ RACE in a classical way (*SI Appendix,* Fig. S8*B*, “Classic 5′ RACE”), the first-round PCR was conducted using synthesized cDNA as input (25-cycle), and second-round PCR used 5 to 10% of the first-round PCR mixture as input (25-cycle). Given the high sequence homology within the first exons of *VIM2*, *VIM3*, and *VIM4,* and the predicted secondary structure between the first exons of *VIM2* and *VIM4* in Cvi, 5% (v/v) DMSO or 1 M betaine was added to each PCR to improve amplification efficiency. Final PCR products were then size-selected via gel electrophoresis, and size-selected products cloned and sequenced by capillary electrophoresis-based Sanger sequencing. To perform 5′ RACE with high-throughput sequencing, libraries were prepared from first-round PCR product via second-round PCR performed with illumina-indexed adapter (*SI Appendix,* Fig. S8*B*, “Targeted 5′ RACE-Seq,” Dataset S7). Prepared libraries were multiplexed and subjected to PE151 sequencing on the Illumina MiniSeq.

Only the 5′ adapter-containing reads from either Sanger or Illumina sequencing (Read 1) were retained for downstream analysis. Adapters were trimmed using *Cutadapt* (v4.1, *-m 20*) (33). The trimmed reads were aligned to *VIM2*, *VIM3*, and *VIM4* genomic sequences—comprising the first exon and 500-bp upstream regions—extracted from the TAIR10 (Col) or Cvi2.0 genomes, using *Bowtie2* (v2.4.2, *--end-to-end --norc*) (34). Only reads aligned without mismatches were selected and subjected to the subsequent analysis and visualization via IGV.

To identify cleavage signatures in the transcripts of *VIM1*, *VIM2*, and *VIM5,* that are presumably mediated by sRNAs derived from the *VIM4*–*VIM2* region in Cvi, modified 5′ RACE protocol that excludes the serial CIP–TAP treatment was performed using DNase I-treated total RNA as an input. 5′ RNA adapter ligation, cDNA synthesis, and PCR amplification were performed subsequently as described above, using primers designed for this targeted 5′ RACE experiment (Dataset S7). After final amplification, amplicons were subjected to PE151 sequencing on the Illumina MiniSeq platform. Trimming and alignment as above.

## Supplementary Material

Appendix 01 (PDF)

Dataset S01 (XLSX)

Dataset S02 (XLSX)

Dataset S03 (XLSX)

Dataset S04 (XLSX)

Dataset S05 (XLSX)

Dataset S06 (XLSX)

Dataset S07 (XLSX)

## Data Availability

All sequencing datasets produced by this article can be accessed through the NCBI SRA (Project No. PRJNA835511) (5). The dataset(s) supporting the conclusions of this article are included within the article, *SI Appendix* and Dataset.
