# Supplementary material for: Genome-wide CG hypomethylation of the Arabidopsis ecotype Cvi linked to structural variation and RNAi at the VIM4–VIM2 locus
Source: Proc Natl Acad Sci U S A. 2026 May 19;123(21):e2603682123. doi: 10.1073/pnas.2603682123 (PMC13213937; doi:10.1073/pnas.2603682123)
Supplement: Supplementary file 1 — Appendix 01 (PDF) [file pnas.2603682123.sapp.pdf]

**Supporting Information for**

# Genome-wide CG hypomethylation of the Arabidopsis ecotype CVI linked to structural variation and RNAi at the *VIM4* – *VIM2* locus

Sang-Yoon Shin[1,2,7], Minsu Park[1,2,3], Jaehoon Lee[2,4], Seunga Lee[2,4], Jennifer M. Frost[5], Robert L. Fischer[6], Yeonhee Choi[2,4] and Chanseok Shin[1,2,3]

Affiliations:

[1]Research Institute of Agriculture and Life Sciences, and Plant Genomics and Breeding Institute, Seoul National University, Seoul, 08826, Korea

[2]Research Center for Plant Plasticity, Seoul National University, Seoul 08826, Korea

[3]Department of Agricultural Biotechnology, Seoul National University, Seoul 08826, Korea

[4]Department of Biological Sciences, Seoul National University, Seoul 08826, Korea

[5]Department of Medical and Molecular Genetics, Great Maze Pond, King's College London, London, United Kingdom

[6]Department of Plant and Microbial Biology, University of California, Berkeley, CA 94720, USA

[7]Present address: Macrogen Inc., Seoul 06221, Korea

**Corresponding authors**
Robert L. Fischer
Email: rfischer@berkeley.edu

Yeonhee Choi
Email: yhc@snu.ac.kr

Chanseok Shin
Email: cshin@snu.ac.kr

**This PDF file includes:**

Supporting text
Figures S1 to S15
SI References

**Other supporting materials for this manuscript include the following:**

Datasets S1 to S7

**Materials and Methods**

**Phase score analysis.** To calculate phase scores for specific genomic regions, all small RNA-Seq datasets from Cvi seed samples were merged into a single BAM file and converted to bedGraph format. Phase scores were then estimated using the following equation (1):

$$Phase\ score = ln\left[\left(1 + 10\ \times \frac{\sum_{i=1}^{10} Pi_i}{1 + \sum U}\right)^{n-2}\right], n > 3,$$

where n is the number of phase-cycle positions occupied by at least one small RNA read within a 10-cycle window, and Pii represents the total number of raw reads for all small RNAs with consolidated start coordinates at phase position i within the window. The calculation was performed using a sliding window with single-nucleotide steps, and scores were assigned to the fifth cycle position in phasing plots. Phase scores were visualized using an in-house R script with manual adjustment.

**Transcriptome Sequencing and analysis** Total RNAs were extracted from FH, AR and GS seeds of Col and Cvi using Trizol (Invitrogen). Each 5 ug of total RNA extracted from same biological batch was used as an input for poly(A) selection using oligo(dT)-conjugated magnetic Dynabeads (Invitrogen) and rRNA depletion carried out using the Ribo Zero Plant kit (Illumina). Two biological replicates for each combination of sample and treatment were prepared, and all transcriptome libraries were constructed in a strand-specific manner using the dUTP-based strand-marking library prep (2). Libraries were sequenced using PE101 on an Illumina HiSeq2500. Reads from Col samples were aligned to TAIR10, and reads from Cvi samples were aligned to its pseudogenome using HISAT2 with the strand-specific option (3). To perform reference-based transcript assembly and measure raw read counts, mapped reads were passed to StringTie (4) and gffcompare (5). FeatureCounts (6) were then used to measure the expression level of genes in TAIR10 and assembled transcripts, and differential gene expression analysis conducted using edgeR (*glmQLFTest*) (38).

**Analysis of publicly available datasets** For ATAC-Seq, datasets were downloaded from the European Nucleotide Archive (ENA, PRJEB54034) and were analyzed as in (7), with modifications indicated below. For PacBio, Cvi's raw PacBio sequencing dataset was downloaded from ENA (PRJEB31147) (8), and the first 24-mer sequence of *VIM2*$_{Col}$ CDS (5′-**ATG**GCGATTGAAACTCAGCTTCCT-3′) was searched against PacBio reads using *Seqkit locate* (v2.3.1) to find *VIM2*$_{Cvi}$- or *VIM4*$_{Cvi}$-encoding read(s) spanning the intergenic region between *VIM2*$_{Cvi}$ and *VIM4*$_{Cvi}$ (9). PacBio reads and short WGS reads found to have Cvi's short intergenic regions between *VIM2*$_{Cvi}$ and *VIM4*$_{Cvi}$ are listed (Dataset S4). For Fig. S10, the list of FPKM-normalized mean expression values were retrieved from GSE118371 (10).

***VIM4* – *VIM2* region identification and comparative sequence analysis** *A. thaliana* ecotype genomes were downloaded either from NCBI (PRJNA1033522)(11) or the China National Center for Bioinformation (PRJCA012695) (12). For genomes downloaded from PRJNA1033522, corresponding GFF3 files were downloaded from EDMOND (https://edmond.mpg.de/dataset.xhtml?persistentId=doi:10.17617/3.AEOJBL). Reference genomes and gene annotation files of *A. lyrata* (Alyrata_384_v2.1) and *A. halleri* (Ahalleri_765_v2.1.0) were downloaded from Phytozome (https://phytozome-next.jgi.doe.gov/), and that of *A. arenosa* was downloaded from NCBI (GCA_905216605.1).

To identify the genomic loci of *VIM2*, *VIM4* and their neighboring genes/TEs across multiple *A. thaliana* ecotype genomes, the gene model under gene IDs shown in Fig. S13 were searched and retrieved from gene annotation files corresponding to each ecotype's genome

assembly (Fig. S13). In parallel, to verify the presence and orientation of homologous *VIM* family genes, *Seqkit locate* (v2.3.1, *-i*, allowing 1-base mismatch per five base) was employed to search for conserved sequences: specifically, the first 60-mer (including the start codon) and the last 47-mer (including the stop codon) of the CDS regions of $VIM1_{Col}$, $VIM2_{Col}$, $VIM3_{Col}$ and $VIM5_{Col}$ were queried against each ecotype's reference genome sequence. In cases where partial gene duplication was suspected (i.e. TueSB30-3 in Fig. 6A) or the gene model was not found from the annotation (i.e. Dog-4 in Fig. S12A, Ket-10 in Fig. S12B), the exon and intron sequences of $VIM2_{Col}$ and $VIM4_{Col}$ were used as BLASTN queries to delineate fragmented or rearranged compartments across the genome. After identifying both boundaries of two *VIM* genes, the sequences of TEs located within the intergenic region between $VIM4_{Col}$ and $VIM2_{Col}$ were queried to the sequence of region covering *VIM4* – *VIM2* IR via BLASTN to determine their presence and genomic coordinates. For *A. thaliana* ecotypes with exceptionally long intergenic regions (i.e. Zin9, Rubezhnoe-1 and ecotypes in the "Madeira" population in Fig. S12) as well as other three *Arabidopsis* species – *A.lyrata*, *A.halleri* and *A.arenosa* (Fig. 6C), all genes/TEs in Araport11 were queried to identify the intergenic region between *VIM4* and *VIM2* (<1E-55 for gene and <1E-10 for TE). Because *VIM2* and *VIM4* were highly similar in terms of sequence homology, we considered the following genomic features in determining the relative position of *VIM2* and *VIM4* in each ecotype : (i) the strand direction and orientation of TEs located in the intergenic region between *VIM2* and *VIM4*, (ii) the presence, position, and genomic orientation of *VIM2*-specific 5′ UTR sequences, (iii) the identity and strand orientation of flanking genes located upstream and downstream of the *VIM4* – *VIM2* IR locus, (iv) sequence variation at the last exon/CDS that was found to be variable between *VIM4* and *VIM2* as described in Fig. 2A (Fig. S12A and B). To check and visualize the synteny across *VIM4* – *VIM2* IR region and its up-/down-stream, *nucmer* (*-maxmatch -c 200 -b 200 -l 50* for Fig. 6 and Fig. S12, *--maxmatch -c 500 -b 200 -l 100* for Fig. S13) and *showcoord* (*-THdlqc*) in MUMmer package for sequence alignment and SyntenyPlotteR for visualization was used (13, 14). Visualization of gene models and TEs was performed using TBtools-II (15).

**Phylogenetic analysis among VIM-homologous proteins encoded in the Brassicales plants**
To identify VIM homologs from 37 plant species belonging to the order *Brassicales*, amino acid sequences of six VIM proteins annotated in TAIR10 were queried against the protein sequence databases of each plant, selecting results with E-values below 1E-100. In total, 180 protein sequences including six from *A. thaliana* (Dataset S6) were subjected to the multiple sequence alignment using MUSCLE5 (16), followed by *trimAl* (*-automated1*) (17) and RAxML (*raxmlHPC-PTHREADS-SSE3 -m PROTGAMMADAYHOFF -# 1000*) (18). The resultant phylogenetic tree was then visualized using MEGA11 (19). Lineages/tribes/families within the order Brassicales are distinguished by colored dots according to the classification from a previous study (20).

## Figures and Legends

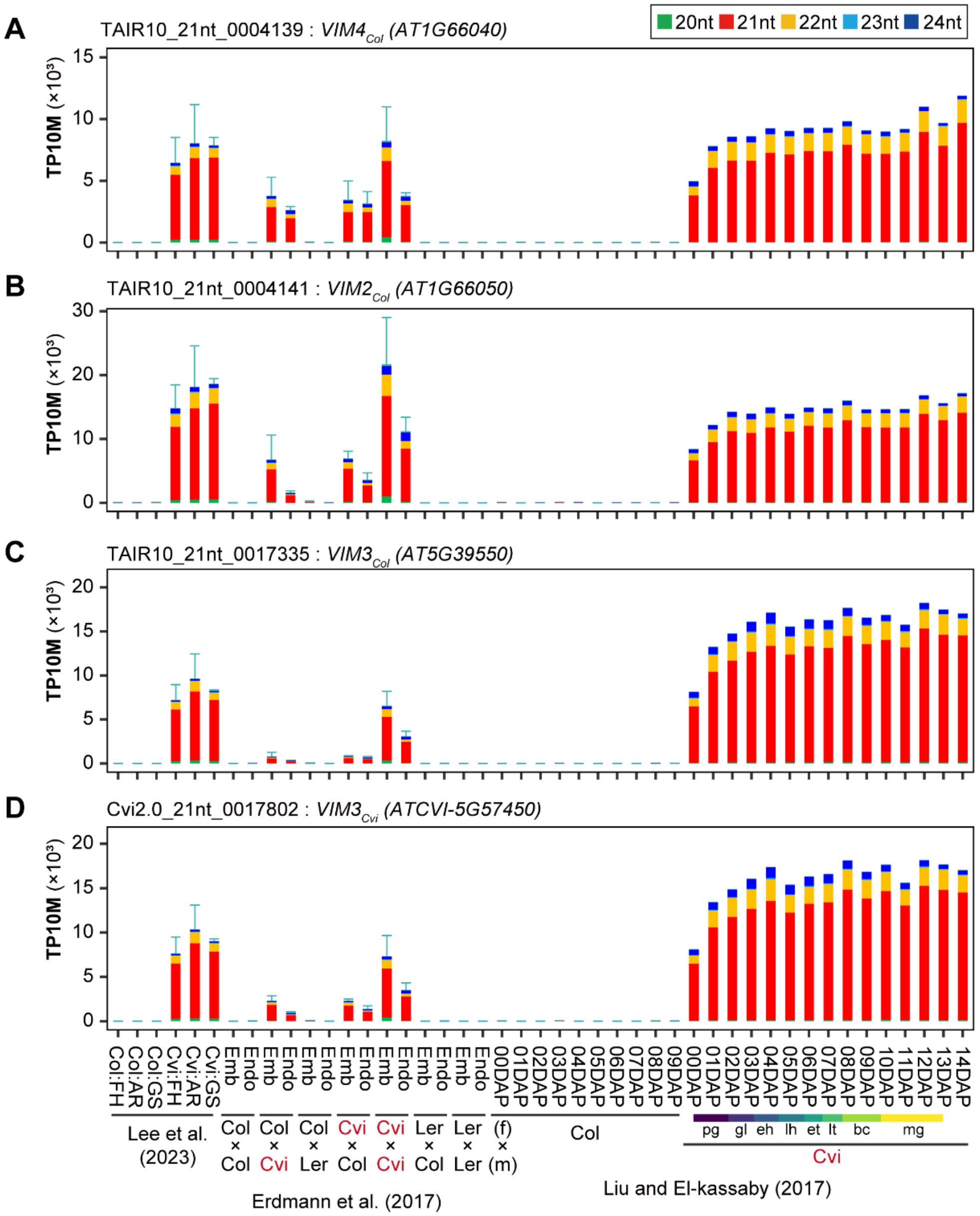


**Fig. S1. Small RNA abundance measured from small RNA clusters located on *VIM* family gene-encoding genomic loci in TAIR10 and Cvi2.0 reference genome.** *(A,B and C)* Spatio-temporal expression pattern and size composition of sRNAs produced from the region spanning 1$^{st}$ exon of three *VIM* family homologs in TAIR10 reference genome. *(D)* Spatio-temporal expression pattern and size composition of sRNAs produced from the region spanning 1$^{st}$ exon of *VIM3* homolog in Cvi2.0 reference genome. The height of the bar graph represents the mean TP10M-normalized sRNA abundance measured across sRNA-Seq replicates, and the error bars represent the standard deviation of TP10M values for the total abundance of 20–24 nt sRNAs across replicates. Each color in the bar graph represents the abundance of each sRNA size. For the datasets from Erdmann et al., the maternal ecotype comes above the “×” sign (“(f)” for female) and the paternal ecotype comes below the “×” sign (“(m)” for male). Datasets produced by Liu and El-Kassaby did not include replicate; therefore, no error bars are drawn. FH; freshly-

harvested, AR; after-ripening, GS; germination-stimulated, DAP; days after pollination, Ler; Lansberg erecta, Emb; embryo, Endo; endosperm, pg; preglobular, gl; globular, eh; early heart, lh; late heart, lt; late torpedo, bc; bent cotyledon, mg; mature green.

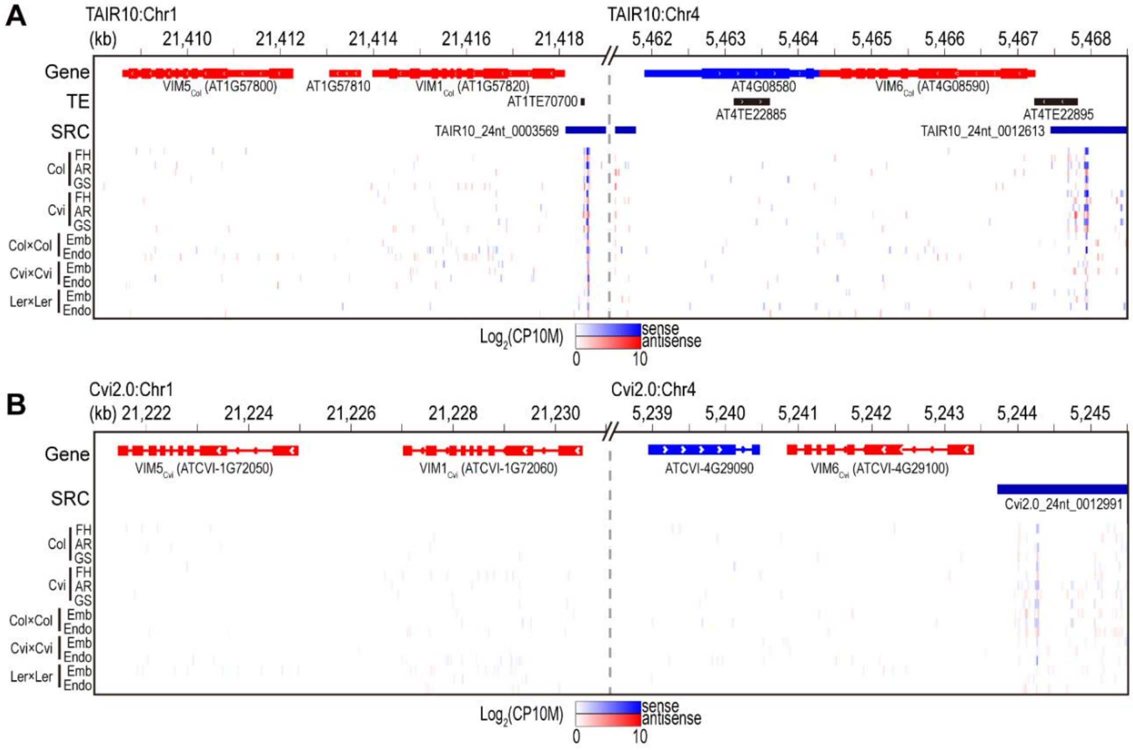


**Fig. S2. Absence of an abundant sRNA population mapped to other three *VIM* family homologs.** *(A and B)* Distribution of sRNAs across three *VIM* family genes-encoding loci on TAIR10 (A) and on Cvi2.0 (B). Compared to the abundant sRNA populations detected from 1st exons of *VIM2*, *VIM3* and *VIM4* homologs, no such abundant sRNAs are observed in the three genic loci encoding the other three *VIM* family homologs, which supports that sRNAs aligned to the *VIM2*, *VIM3* and *VIM4* homologs are produced from those loci. $Log_2$-transformed CP10M-normalized sRNA abundance is represented as a heatmap. For sRNA abundance heatmap, sRNAs mapped to a genome sequence in sense are colored in blue, and sRNAs mapped in antisense are colored in red. FH:freshly harvested, AR:after-ripening GS:germination-stimulated, Emb:embryo, Endo:endosperm.

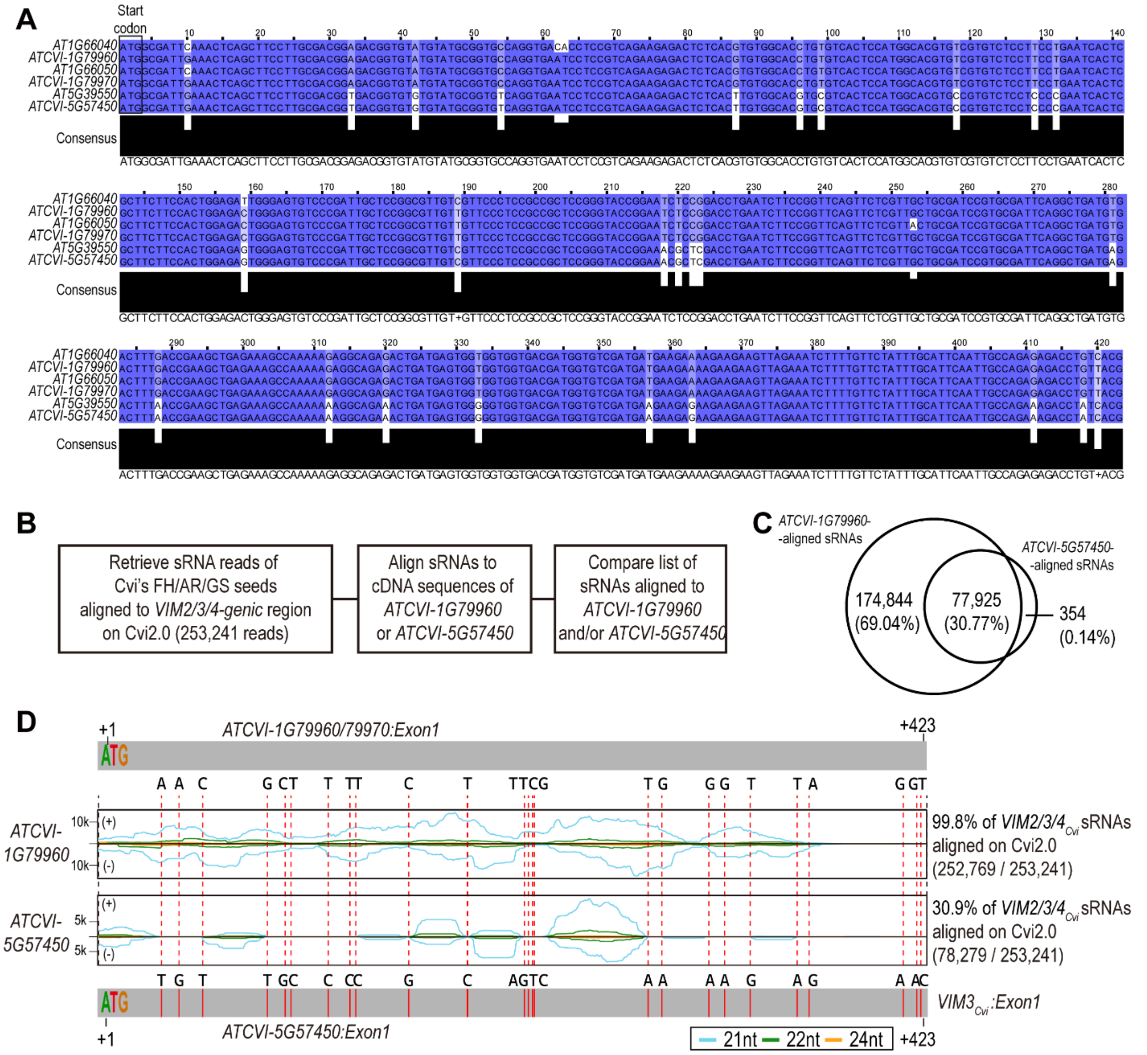


**Fig. S3. *VIM3$_{Cvi}$* is not responsible for sRNA production.** *(A)* High sequence similarity between the coding sequences in the 1$^{st}$ exon of *VIM2/3/4* homologs of Col (AT1G66050, AT5G39550 and AT1G66040) and Cvi (ATCVI-1G79970, ATCVI-5G57450 and ATCVI-1G79960). *(B)* Schematics of re-alignment analysis of the Cvi's *VIM2/3/4*-mapped sRNAs. *(C)* Numbers of sRNAs that are retrieved from *VIM2/3/4$_{Cvi}$*-encoding loci and are aligned to the cDNA sequences of ATCVI-1G79960 and/or ATCVI-5G57450. *(D)* Read distribution of 21-nt, 22-nt and 24-nt sRNAs mapped across 1$^{st}$ exon region of ATCVI-1G79960 and/or ATCVI-5G57450. The height of the line plot indicates the raw base coverage of aligned sRNAs. Positions showing different sequence between ATCVI-1G79960 and ATCVI-5G57450 are indicated by dashed or solid lines in red. ATCVI-1G79960-biased alignment of sRNAs across these ATCVI-1G79960 sequence-specific region strongly suggests that these sRNAs are unlikely to originate from the genic region of ATCVI-5G57450.

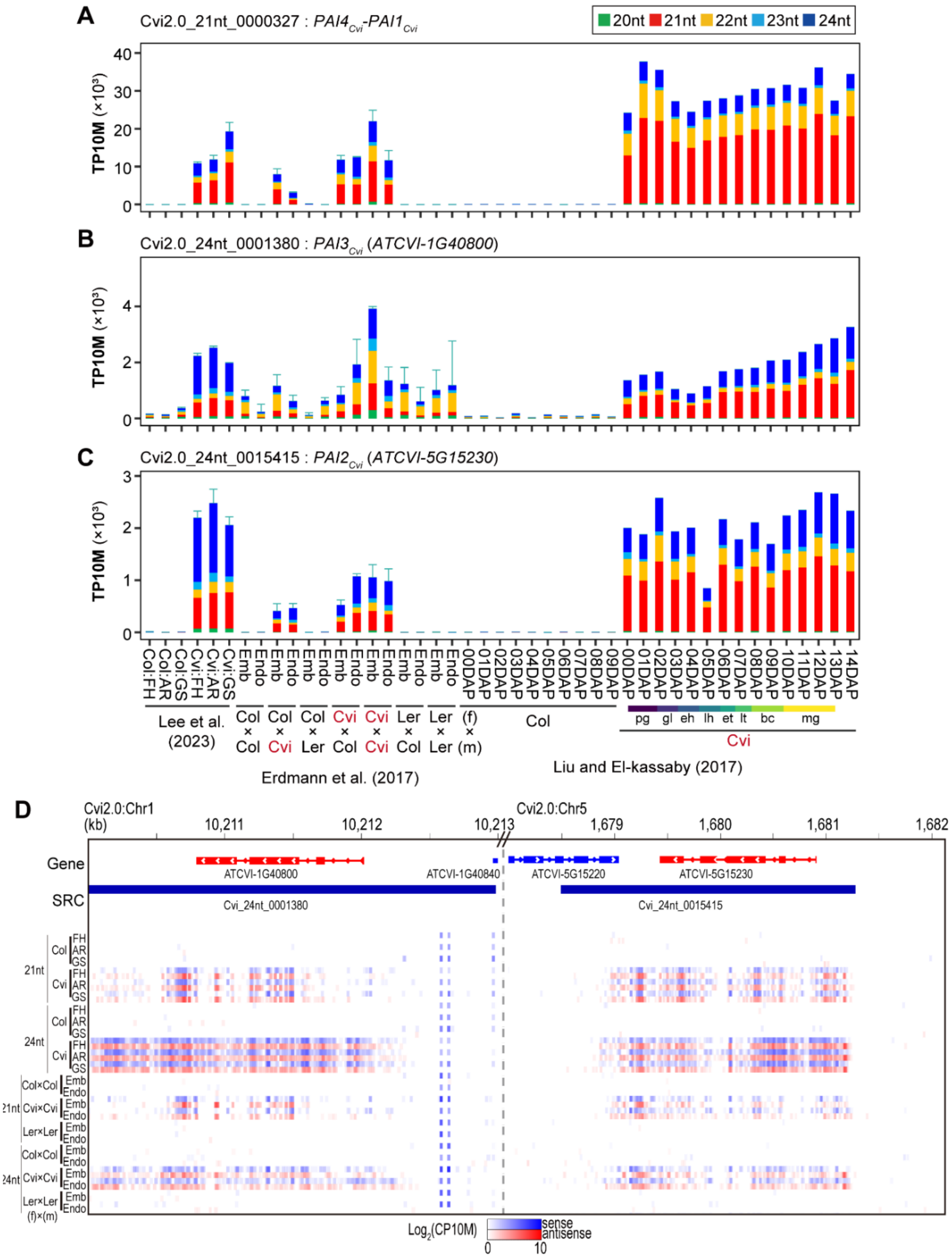


**Fig. S4. Small RNA abundance measured from small RNA clusters located on *PAI* family gene-encoding genomic loci in Cvi2.0 reference genome.** *(A,B and C)* Spatio-temporal expression pattern and size composition of sRNAs produced from the region encoding *PAI* family genes in Cvi2.0 reference genome. The height of the bar graph represents the mean TP10M-normalized sRNA abundance measured across sRNA-Seq replicates, and the error bars

represent the standard deviation of TP10M values for the total abundance of 20–24 nt sRNAs across replicates. Each color in the bar graph represents the abundance of each sRNA size. For the datasets from Erdmann et al., the maternal ecotype comes above the "×" sign ("(f)" for female) and the paternal ecotype comes below the "×" sign ("(m)" for male). Datasets produced by Liu and El-Kassaby did not include replicate; therefore, no error bars are drawn. FH; freshly-harvested, AR; after-ripening, GS; germination-stimulated, DAP; days after pollination, Ler; Lansberg erecta, Emb; embryo, Endo; endosperm, pg; preglobular, gl; globular, eh; early heart, lh; late heart, lt; late torpedo, bc; bent cotyledon, mg; mature green. *(D)* sRNA distribution across Cvi's genomic region encoding two *PAI* family genes (left; $PAI3_{Cvi}$, right; $PAI2_{Cvi}$). $Log_2$-transformed CP10M-normalized sRNA abundance is represented as a heatmap. For sRNA abundance heatmap, sRNAs mapped to a genome sequence in sense are colored in blue, and sRNAs mapped in antisense are colored in red. SRC; small RNA cluster.

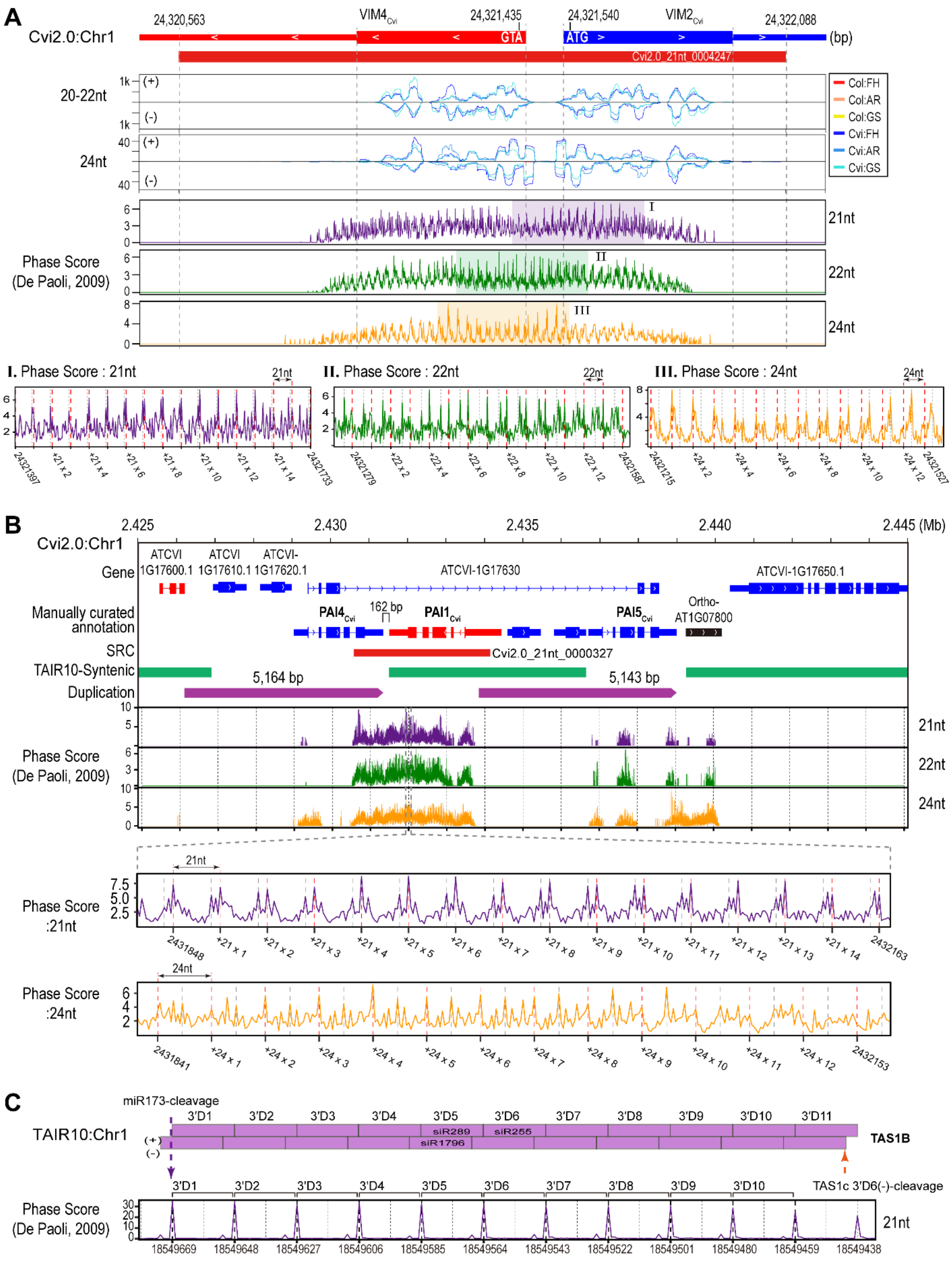


**Fig. S5. Small RNAs are produced from the *VIM4$_{Cvi}$* – *VIM2$_{Cvi}$* region and the *PAI4$_{Cvi}$* – *PAI1$_{Cvi}$* region in a phased manner.** *(A)* Distribution of *VIM4$_{Cvi}$* – *VIM2$_{Cvi}$* region-derived sRNAs and an estimated phase score. Each colored line represents six different seed samples prepared and analyzed in this study. Phase scores for each size of small RNAs. Violet, green and orange-colored lines represent 21nt, 22nt and 24nt small RNA phase scores, respectively. The phase score is calculated after pooling all small RNA datasets generated from samples at the Freshly

Harvested (fully matured green; FH), After Ripening (AR) and Germination-Stimulated (GS) seed stages of Cvi seed maturation. Region with the highest Phase Scores for each small RNA size (highlighted by colored background in each track of phase score and marked as I, II and III) are shown below with a close-up view. *(B)* Phase scores estimated for each size of small RNAs across the *PAI4*$_{Cvi}$ – *PAI1*$_{Cvi}$ region. For *(A)* and *(B)*, the primary (most distinct) phasing patterns are indicated by red-dashed lines, while the secondary patterns are marked by grey-dashed lines. *(C)* The phase score and the patterns observed at *AtTAS1B*, one of well-known trans-acting siRNA-generating regions. After the *AtTAS1B* transcript is cleaved by ath-miR173, 21-nt phased siRNAs are produced downstream of the cleavage site, with the phasing initiated at the point of miR173-mediated cut.

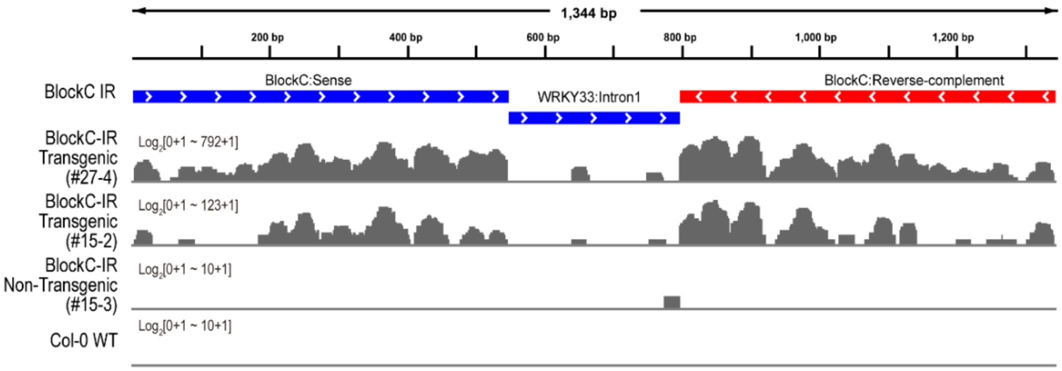


**Fig. S6. Small RNA read distribution across *Block C* IR.** sRNA reads from sRNA-Seq datasets (SRR6456283, SRR6456284, SRR6456285, SRR8069224) aligned to the Block C IR interleaved by *WRKY33*'s first intron. Similar with the sRNA distribution in Fig. 2B and C, sRNAs are mainly produced from a stem-forming palindrome region and only few sRNAs are mapped to a loop region.

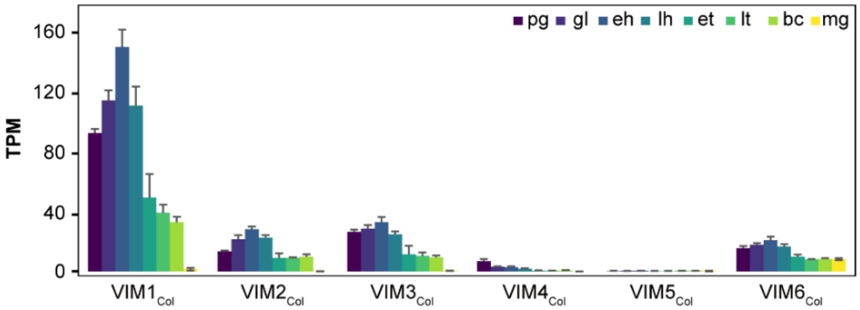


**Fig. S7. Time-course expression of six *VIM$_{Col}$* genes in embryo across development stages.** TPM-normalized transcript abundances of six *VIM$_{Col}$* genes measured from developing embryos of the Col-0. Values for the normalized expression values and standard deviations are referred from the published datasets provided by Hofmann et al..

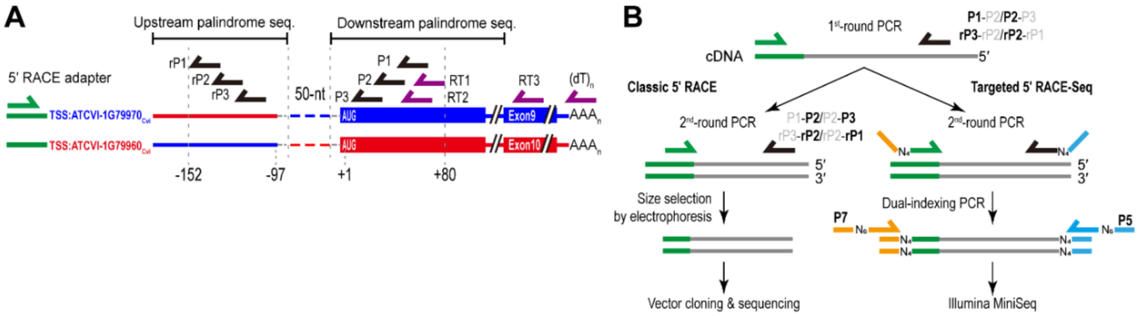


**Fig. S8. Schematic representation of procedure for 5′ RACE.** *(A)* Schematics of primer positions across *VIM2*/*VIM4* homologs' gene model. Arrows colored in violet represent primers for reverse transcription, and arrows colored in black represent 5′ RACE primers. *(B)* Schematics of procedure for classic 5′ RACE, and an modified version of 5′ RACE for an NGS-based high-throughput analysis.

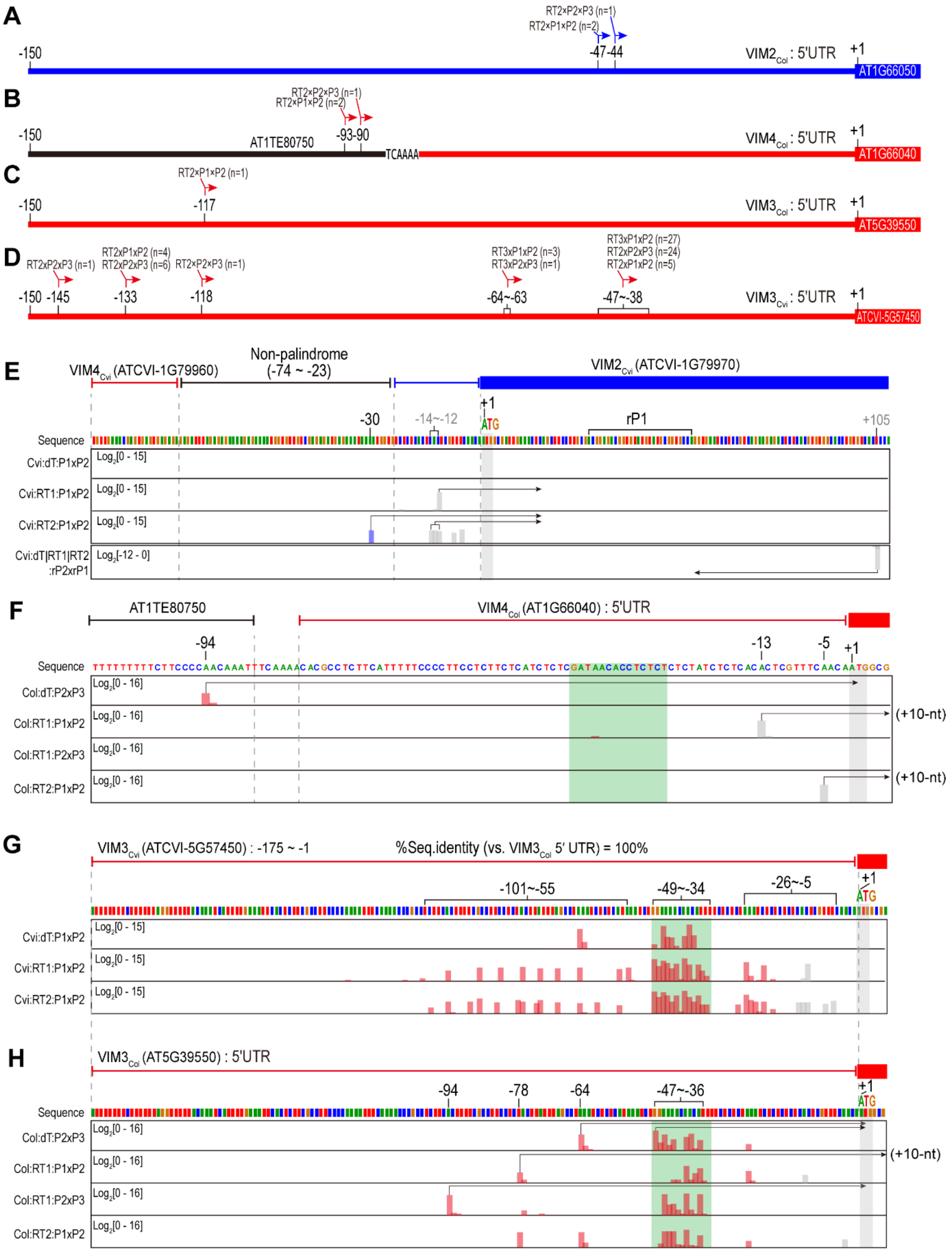

**Fig. S9. Results of 5′ RACE and 5′ RACE-Seq analyzed from Col and Cvi samples.** *(A-D)* Schematic representation of the 5′ RACE-profiled 5′ end position and the orientation of sequenced tags for *VIM2Col* (A), *VIM4Col* (B), *VIM3Col* (C) and *VIM3Cvi* (D). Label on each identified position represents primers (separated by a cross mark) used in reverse transcription (“RT#”), first-round PCR and second-round PCR, respectively. Position and direction of arrow represents 5′ end and orientation of sequenced 5′ RACE tag, respectively. *(E-H)* 5′ end position of

sequenced tags identified by targeted 5′ RACE-Seq and being aligned to the upstream of *VIM2*$_{Cvi}$ (E), *VIM4*$_{Col}$ (F), *VIM3*$_{Cvi}$ (G) and *VIM3*$_{Col}$ (H). The peak height represents $\log_2$-transformed raw number of tags having same 5′ end position, and peak color represent the sequenced tags whose orientation matches to the transcription direction of each corresponding genes, respectively. Label on the left of track represents primers (separated by a cross mark) used in reverse transcription (“RT#”), 1$^{st}$-round PCR and 2$^{nd}$-round PCR, respectively. Green boxes indicate the region that is considered as the major transcription start site (G, H) or contains sequence homologous to that on the major transcription start site of other *VIM2/3/4* genes (F).

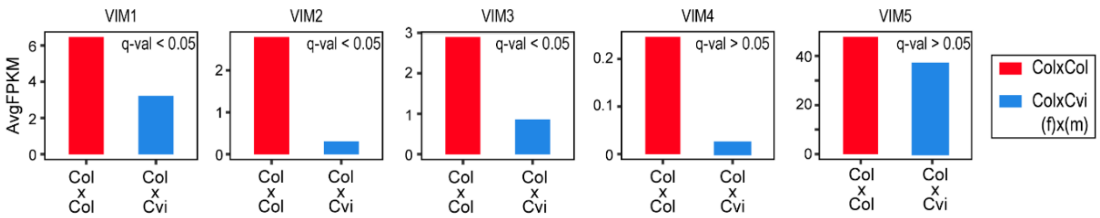


**Fig. S10. Decrease of *VIM* family genes' transcript level in the endosperm of a hybrid between Col-0 and Cvi-0 compared to those in the endosperm of a homozygous Col-0.** FPKM-normalized mean expression of five *VIM* family genes retrieved from GSE118371. Standard deviation for mean FPKM value is not represented due to the absence of corresponding dataset in GSE118371. "q-val" indicates corrected *p*-value provided from the Cuffdiff result conducted by Pignatta et al. (2018).

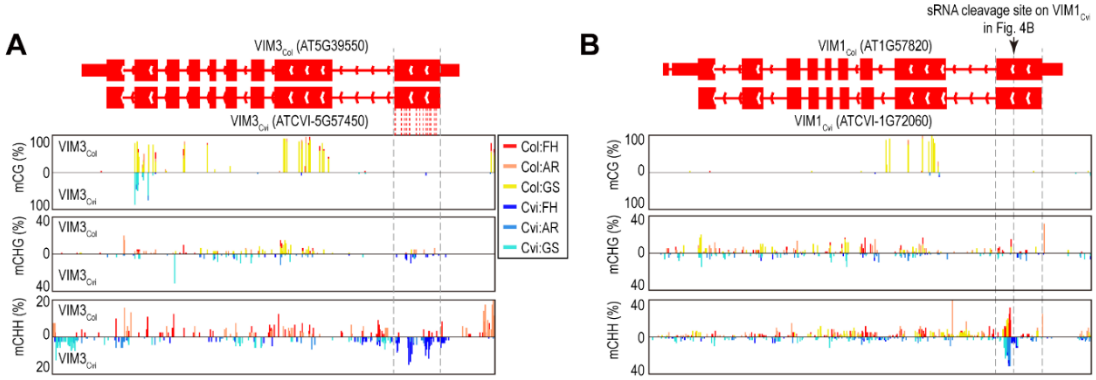


**Fig. S11. *VIM2/4$_{Cvi}$*-derived sRNA may be involved in a *VIM2/3/4* homologs-specific non-CG DNA methylation in Cvi.** *(A and B)* DNA methylation level distribution on three cytosine methylation contexts across the *VIM3$_{Col}$* and *VIM3$_{Cvi}$* (A), and across the *VIM1$_{Col}$* and *VIM1$_{Cvi}$* (B). Red-colored dotted lines in (A) represent sequences on 1$^{st}$ exon that are different from 1$^{st}$ exon sequence of *VIM2$_{Cvi}$* or *VIM4$_{Cvi}$* represented in Fig. S3. Arrow in (B) indicates cleavage site on *VIM1$_{Cvi}$* that is predicted to be targeted by *VIM2/4*-derived sRNAs and is identified by the 5′ RACE-Seq (Fig. 4B).

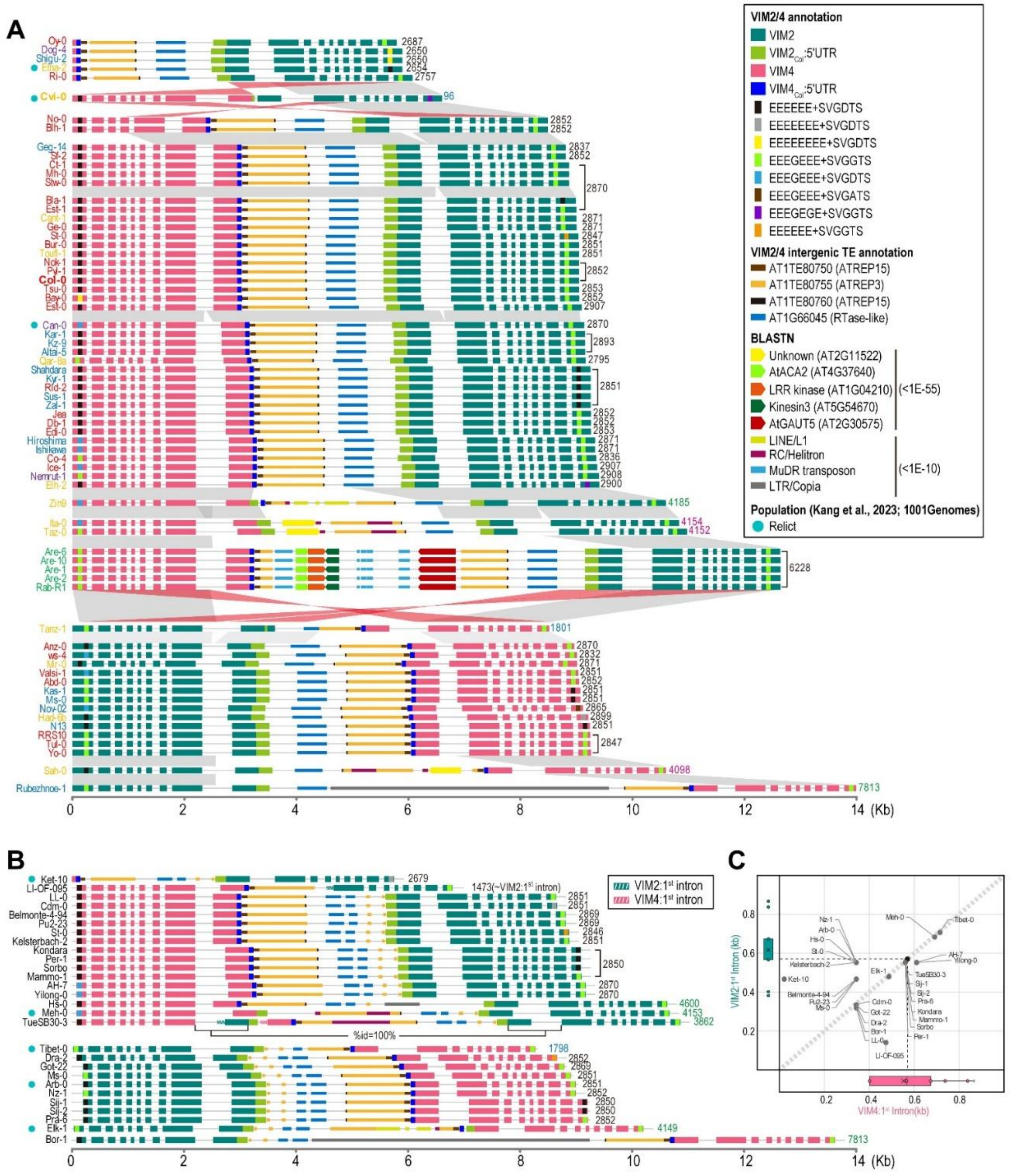


**Fig. S12. Sequence conservation and variation on *VIM4* – *VIM2* IR region between *A. thaliana* accessions.** *(A and B)* Schematic diagrams of the *VIM4* – *VIM2* IR structures of each *A.thaliana* accessions found from *de novo* genome assemblies provided by Lian et al. (A) or Kang et al. (B). Exons of *VIM2* and *VIM4* being annotated by Lian et al. are represented by turquoise green and pink, respectively. 5′ UTR sequences of $VIM2_{Col}$ and $VIM4_{Col}$ are represented by light green and red, respectively. Colored boxes between *VIM2* and *VIM4* represent sequences encoding transposable elements and those homologous to gene fragments. The numbers written to the right of the *VIM4* – *VIM2* IR structure for each accession indicate the distance between the start codons of *VIM2* and *VIM4* for that accession. The gray or pale-red links between the boxes represent homologous relationships analyzed by nucmer in MUMmer package. Colors of

accession names labeled on the left of each lines in (A) represent the genetic classification of each accession provided by Lian et al (2024), which is “Europe” (red), “Madeira” (green), “Asia” (blue), “Africa” (orange) and “Admixed” (violet). Relict accessions are labeled with skyblue dot on the left of each accession name. **C** Pairwise distribution of the 1$^{st}$ intron length of *VIM2* and *VIM4* homologs among 27 *A. thaliana* accessions analyzed by Kang et al. (2023). Black dot indicates the median of *VIM3*’s 1$^{st}$ intron lengths of 27 accessions.

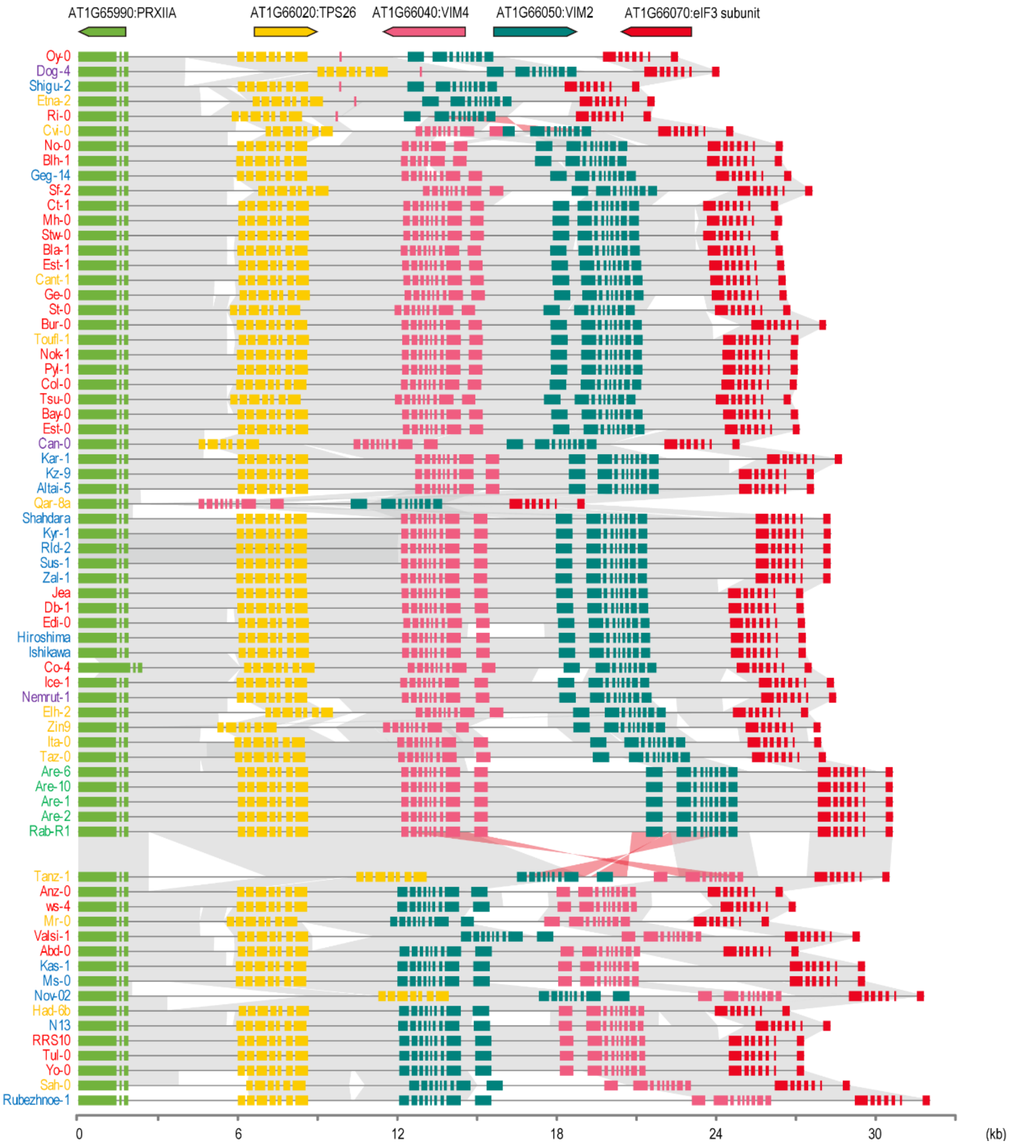


**Fig. S13. The local syntenic order of genes across *VIM4* – *VIM2* palindrome-located region and its upstream and downstream regions among 69 *A. thaliana* ecotypes.** The local syntenic gene/TE order in the region between two genes, AT1G65990 and AT1G66070, conserved in all ecotypes. Exons of five genes are represented by colored rectangles. The gray links between the boxes represent homologous relationships. The pale-red links between the boxes represent inverted homologous relationship. The font color of ecotype names indicates the genetic classification of each ecotype performed by Lian et al. (2024), which is “Europe” (red), “Madeira” (green), “Asia” (blue), “Africa” (orange) and “Admixed” (violet).

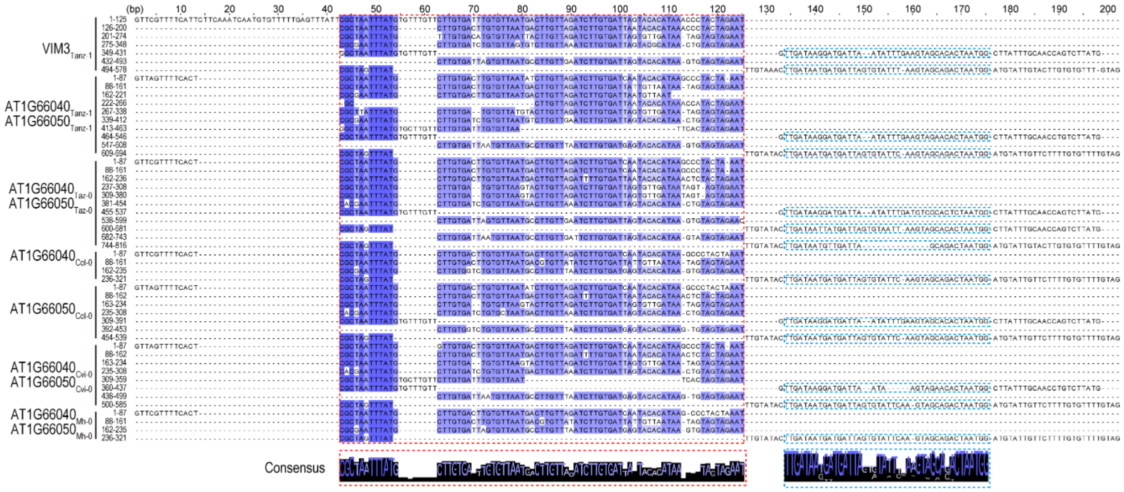

**Fig. S14. Variation in length of the first introns of *VIM2, VIM3* and *VIM4* is caused by the copy number variation of a highly repetitive sequence motif.** Two repetitive sequence domains are boxed with red- and skyblue-dotted lines, respectively.

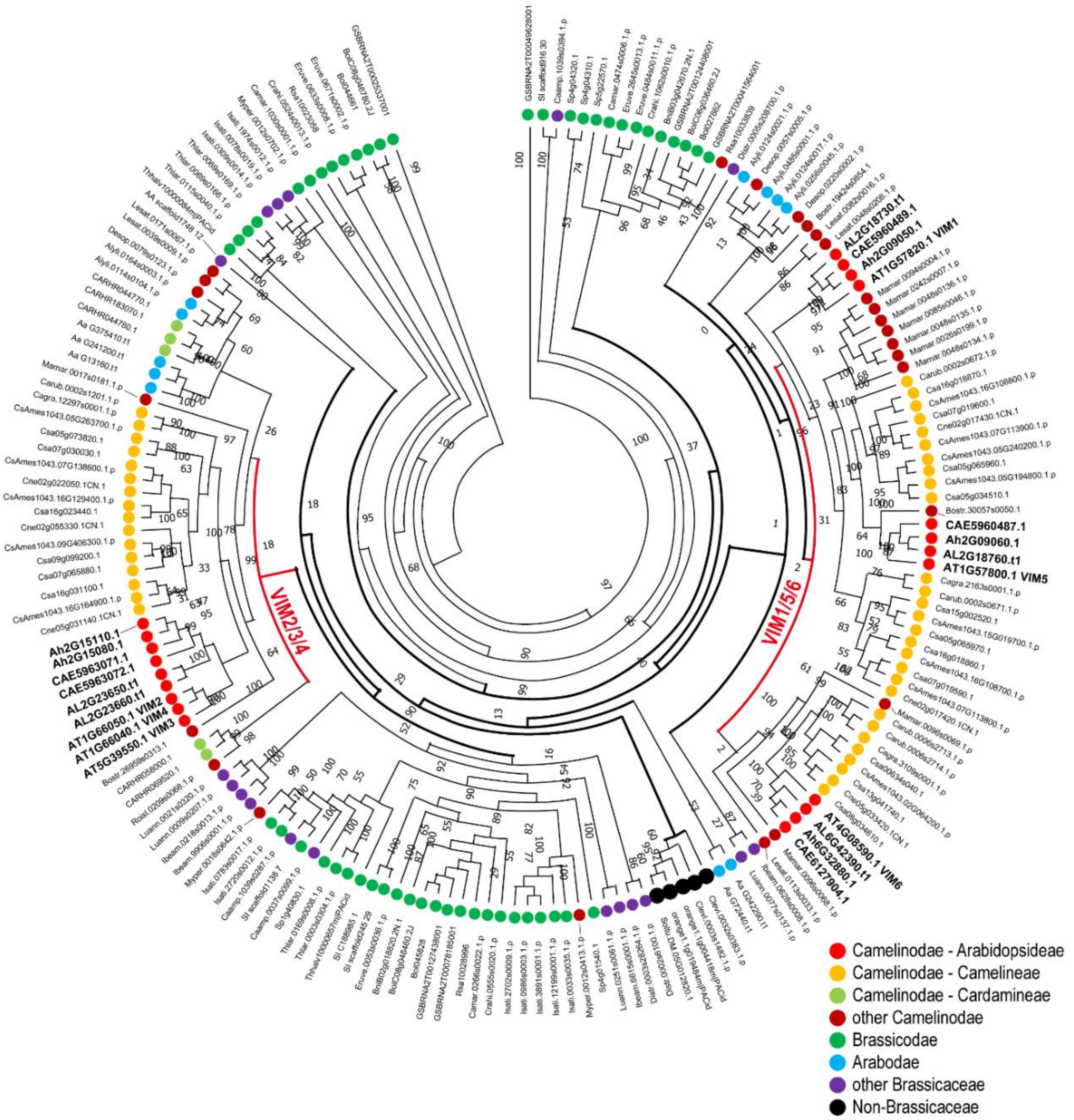


**Fig. S15. Phylogenetic tree representation of *VIM* family-homologous genes in 39 plants.** Phylogenetic relationship between *VIM* family-homologous genes identified from 39 plants. Protein IDs in bold represent *VIM* family genes of plants in genus *Arabidopsis*. Colored dots represent lineages/tribes/families within (37 plants)) or outside (*C. sinensis* and *S. tuberosum*) of the order Brassicales that are referred from previously classification suggested by Hendriks et al. (2023).

**Dataset S1. ShortStack-annotated small RNA clusters associated with VIM or PAI family genes annotated on TAIR10 genome.**

**Dataset S2. ShortStack-annotated small RNA clusters associated with VIM or PAI family genes annotated on Cvi2.0 genome.**

**Dataset S3. GFF3-formatted locations of *PAI1*, *PAI4* and *PAI5* gene models on Cvi2.0 genome described in Figure 1D.**

**Dataset S4. WGS reads spanning intergenic region between *VIM4* and *VIM2* in Cvi2.0 genome.**

**Dataset S5. The length of the first intron of *VIM2*, *VIM4* and *VIM3*.**

**Dataset S6. List of VIM-homologous proteins identified from 38 plant species belonging to the order Brassicales.**

**Dataset S7. Oligonucleotides used in 5′ RACE experiment.**

**SI References**


1. E. De Paoli *et al.*, Distinct extremely abundant siRNAs associated with cosuppression in petunia. *RNA* **15**, 1965-1970 (2009).
2. S. Zhong *et al.*, High-throughput illumina strand-specific RNA sequencing library preparation. *Cold Spring Harb Protoc* **2011**, 940-949 (2011).
3. D. Kim, J. M. Paggi, C. Park, C. Bennett, S. L. Salzberg, Graph-based genome alignment and genotyping with HISAT2 and HISAT-genotype. *Nat Biotechnol* **37**, 907-915 (2019).
4. G. Pertea, M. Pertea, GFF Utilities: GffRead and GffCompare. *F1000Res* **9** (2020).
5. M. Pertea, D. Kim, G. M. Pertea, J. T. Leek, S. L. Salzberg, Transcript-level expression analysis of RNA-seq experiments with HISAT, StringTie and Ballgown. *Nat Protoc* **11**, 1650-1667 (2016).
6. Y. Liao, G. K. Smyth, W. Shi, The R package Rsubread is easier, faster, cheaper and better for alignment and quantification of RNA sequencing reads. *Nucleic Acids Research* **47** (2019).
7. T. Srikant *et al.*, Canalization of genome-wide transcriptional activity in Arabidopsis thaliana accessions by MET1-dependent CG methylation. *Genome Biol* **23**, 263 (2022).
8. W. B. Jiao, K. Schneeberger, Chromosome-level assemblies of multiple Arabidopsis genomes reveal hotspots of rearrangements with altered evolutionary dynamics. *Nat Commun* **11**, 989 (2020).
9. W. Shen, S. Le, Y. Li, F. Hu, SeqKit: A Cross-Platform and Ultrafast Toolkit for FASTA/Q File Manipulation. *PLoS One* **11**, e0163962 (2016).
10. D. Pignatta, K. Novitzky, P. R. V. Satyaki, M. Gehring, A variably imprinted epiallele impacts seed development. *PLoS Genet* **14**, e1007469 (2018).
11. Q. Lian *et al.*, A pan-genome of 69 Arabidopsis thaliana accessions reveals a conserved genome structure throughout the global species range. *Nat Genet* (2024).
12. M. Kang *et al.*, The pan-genome and local adaptation of Arabidopsis thaliana. *Nat Commun* **14**, 6259 (2023).
13. G. Marcais *et al.*, MUMmer4: A fast and versatile genome alignment system. *PLoS Comput Biol* **14**, e1005944 (2018).
14. S. Quigley, J. Damas, D. M. Larkin, M. Farre, syntenyPlotteR: a user-friendly R package to visualize genome synteny, ideal for both experienced and novice bioinformaticians. *Bioinform Adv* **3**, vbad161 (2023).
15. C. Chen *et al.*, TBtools-II: A "one for all, all for one" bioinformatics platform for biological big-data mining. *Mol Plant* **16**, 1733-1742 (2023).
16. R. C. Edgar, MUSCLE: multiple sequence alignment with high accuracy and high throughput. *Nucleic Acids Res* **32**, 1792-1797 (2004).
17. S. Capella-Gutierrez, J. M. Silla-Martinez, T. Gabaldon, trimAl: a tool for automated alignment trimming in large-scale phylogenetic analyses. *Bioinformatics* **25**, 1972-1973 (2009).
18. A. Stamatakis, RAxML version 8: a tool for phylogenetic analysis and post-analysis of large phylogenies. *Bioinformatics* **30**, 1312-1313 (2014).
19. K. Tamura, G. Stecher, S. Kumar, MEGA11: Molecular Evolutionary Genetics Analysis Version 11. *Mol Biol Evol* **38**, 3022-3027 (2021).
20. K. P. Hendriks *et al.*, Global Brassicaceae phylogeny based on filtering of 1,000-gene dataset. *Curr Biol* **33**, 4052-4068 e4056 (2023).
